# Satisfied with the worst health outcomes or unsatisfied with the best: explaining the divergence between good patient-reported outcomes and low satisfaction and vice versa among knee arthroplasty patients – a retrospective cohort study

**DOI:** 10.1186/s13018-025-05507-7

**Published:** 2025-01-23

**Authors:** Lukas Schöner, Viktoria Steinbeck, Reinhard Busse, Carlos J. Marques

**Affiliations:** 1 Department of Health Care Management, School of Economics and Management, Technical University Berlin, (Secretariat H80) Strasse des 17 Juni 135, 10623 Berlin, Germany; 2 Department of Performance, Neuroscience, Therapy, and Health, Institute of Interdisciplinary Exercise Science and Sports Medicine, Medical School Hamburg, University of Applied Sciences and Medical University, Am Kaiserkai 1, 20457 Hamburg, Germany

**Keywords:** Patient-reported outcome, Patient satisfaction, Value-based healthcare, Knee arthroplasty, Expectation management, Quality measurement

## Abstract

**Objectives:**

Total knee arthroplasty (TKA) is an effective treatment for patients with end-stage knee osteoarthritis but some patients exhibit a discrepancy between patient-reported outcomes (PROs) and patient satisfaction (PS). This study aims to identify predictors for patients reporting unfavorable PROs but high PS and vice versa.

**Materials and methods:**

This retrospective cohort study categorized patients from nine German hospitals into four groups based on (i) whether they achieved a minimal clinically important difference (MCID) in knee functionality, measured with a joint-specific PRO from admission to 12-month post-surgery; and (ii) whether they were satisfied at 12 months post-surgery. The groups were (A) Satisfied Achievers (satisfied, MCID reached), (B) Dissatisfied Achievers (not satisfied, MCID reached), (C) Satisfied Non-Achievers (satisfied, MCID not reached) and (D) Dissatisfied Non-Achievers (not satisfied, MCID not reached). Exploratory analyses were performed to understand differences between the four groups using chi-squared tests and ANOVA. Multinomial logistic regression models were conducted to identify predictors for the allocation of patients in groups.

**Results:**

A total of 1546 knee arthroplasty patients with a mean age of 65.9 years, 54.1% female, were included. 1146 (74.1%) patients were Satisfied Achievers, 131 (8.5%) were Dissatisfied Achievers, 141 (9.1%) were Satisfied Non-Achievers, and 128 (8.3%) Dissatisfied Non-Achievers. The results showed that higher improvements in health-related quality of life, pain and fatigue symptoms significantly decreased the likelihood of being a Dissatisfied Achiever and a Satisfied Non-Achiever. Comorbidities of blood circulation, chronic back pain or diabetes increased the likelihood of being a Dissatisfied Achiever, while depression decreased the likelihood of being a Satisfied Non-Achiever.

**Conclusion:**

Addressing individual health concerns, e.g. through expectation management, and assessing alternative treatment options might improve satisfaction in line with functional improvements. A closer evaluation at which physical impairment level surgery is beneficial could help to improve the care of Satisfied Non-Achievers.

**Supplementary Information:**

The online version contains supplementary material available at 10.1186/s13018-025-05507-7.

## Introduction

Total knee arthroplasty (TKA) is among the most effective orthopedic treatments for patients with end-stage knee osteoarthritis, who no longer benefit from conservative treatment [1, 2]. Most patients benefit from TKA in the form of improved health-related quality of life (HRQoL), decreased pain, and improvement in function and activities of daily living [3]. However, there is a percentage of patients remaining unsatisfied with TKA [4] – ranging from 12.7% in the US, 8.3% in the UK to 8% in Germany [5–7]. Patient satisfaction is an important patient-centered outcome that is frequently measured with patient-reported outcome measures (PROMs) to assess treatment success from the patient’s perspective [8].

PROMs globally gain attention in orthopedics as they complement common outcome metrics with health outcomes from the patient’s perspective. This is reflected for instance by the growing integration of PROMs into registries (e.g. in Sweden, England, Canada and France) [9], national quality assurance [10], clinical care pathways [11, 12] and value-based procurement [13]. Simultaneously, organizations like the International Consortium for Health Outcome Measurement (ICHOM) and the Organization for Economic Co-operation and Development (OECD) set out recommendations for the assessment of PROMs in TKA in recent years [14, 15].

Despite the intuitive assumption that favorable patient-reported outcomes (PROs) go hand in hand with high patient satisfaction and vice versa, Black et al., however, pointed out that better health does not always mean better experience levels and, hence, it is relevant to understand patients who report positive experiences even though they have not improved in their health status and vice versa [16]. The authors showed only a weak positive correlation between patient experiences and PROs, with PROs increasing patients’ experience ratings by 10%, and patient experiences improving PROs by 3% for the English hip and knee replacement population.

While some studies in the last years were conducted on significant predictors of patient satisfaction or dissatisfaction [5, 8, 17–19], to the best of our knowledge, no studies were published that cluster patients according to their satisfaction and PROMs, as suggested in the study by Klem et al. [20], and identify predictors showcasing which aspects make patients more or less likely to belong to a patient cluster that is satisfied.

The insights into the characteristics of different clusters and their predictors could help physicians during the preoperative clinical shared decision process e.g. to modulate patients’ expectations concerning TKA, thus potentially leading to higher patient satisfaction after surgery. Hence, we aimed to identify predictive characteristics for patients reporting improvements in physical function of the knee from admission to 12-months post-surgery below a clinically meaningful level but reported being satisfied with the surgery 12 months post-surgery and vice versa.

## Materials and methods

### Study design

This retrospective cohort study is a secondary data analysis of the data from the ‘PROMoting Quality’ trial [21]. PROMoting Quality tested the cost-effectiveness of a PROM-based monitoring and alert intervention following hip or knee replacement surgery [22]. Detailed information on the study design of the PROMoting Quality trial was published previously [23]. The trial was registered in the German Trial Register under DRKS00019916 and was approved by the ethics committee of the Charité - Universitätsmedizin, Berlin (EA4/ 169/19). Written informed consent was retrieved from all participants.

This work has been reported in line with the STROCSS criteria [24]. The dataset collected as part of PROMoting Quality covers a wide range of patient characteristics, patient-reported outcomes and clinical indicators.

### Patients

**Fig. 1:**
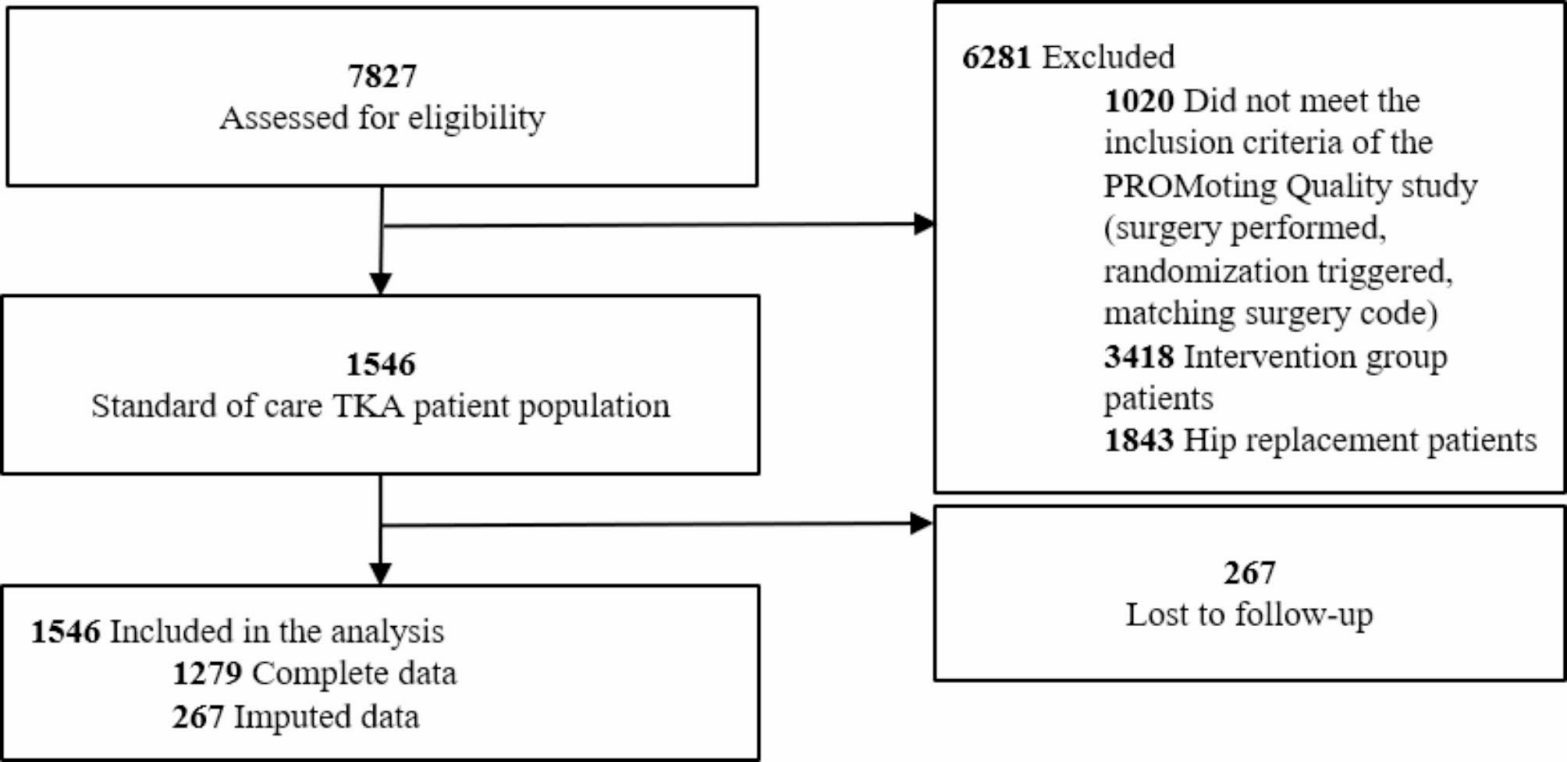
Study population flow. TKA – Total knee arthroplasty

For the present work, data of patients who were admitted for TKA and randomized to the control group was analyzed, as those patients received the German TKA standard of care. This enabled a broader generalizability through increased external validity. Inclusion criteria were adult patients with an elective primary TKA matching a predefined set of surgery codes. Exclusion criteria were patients undergoing hip replacement surgery, TKA patients randomized in the intervention group, emergency cases, patients classified under the American Society of Anesthesiologists (ASA) categories 4–6 (i.e., patients with a severe life-threatening disease, moribund patients who are not expected to survive without an operation within 24 h, brain-dead patients), and patients with lack of direct or indirect access to an Email-account. The selection flow (including patients in the hip replacement group) is shown in Fig. 1. Data of 1546 patients were included in the analyses.

### Patient satisfaction

Patient satisfaction was measured on a five-level likert scale at 12-months post-surgery. The patients were asked, whether they were satisfied with the results of the TKA 12-month post-surgery. The answer levels were “very satisfied” (5), “satisfied” (4), “neither satisfied nor unsatisfied” (3), “unsatisfied” (2), “very unsatisfied” (1).

### Patient-reported outcome measures (PROMS)

Patients received the German versions of the following PROMs at admission to the hospital and at 12-months post-surgery:

- The EuroQol five dimensions five levels questionnaire (EQ-5D-5L) and EuroQol visual analogue scale (EQ-VAS) to measure health-related quality of life (HRQoL). Based on the German value set [25], patients can achieve a health index score that ranges from − 0.661 to 1.0. The VAS values for HRQoL range between 0 and 100, with higher values indicating higher levels of HRQoL.
- The Knee injury and Osteoarthritis Outcome Score Physical Function Short-form (KOOS-PS) was used to measure physical function. The scores range from 0 to 100, with lower values indicating lower physical impairment [26].
- The analogue pain questionnaire, a cumulative score based on the pain in the hip and knee on the right and left side and lower back, with zero indicating no pain and 10 indicating the highest perceived pain.
- And the Patient Reported Outcomes Measurement Information System (PROMIS) Short Form v1.0 - fatigue 4a (PROMIS-SF-F) [27] and depression 4a (PROMIS-SF-D) [28]. The patients can achieve a score that ranges from 33.7 to 75.8 and 41 to 79.4, respectively, with lower values indicating lower levels of fatigue and depression symptoms.

We defined a PRO as the result of PROMs, i.e. PROM scores at hospital admission (i.e. “admission PRO”), at 12-month follow-up (i.e. “12-months PRO”), and the change between hospital admission and 12-month follow-up (i.e. “PRO change”). In the context of this study, we examined the association between satisfaction and PROs.

### Statistical analysis

First, descriptive statistics were run for the patients who were admitted for TKA. Secondly, pearson’s correlation coefficients between satisfaction levels and the selected PROs were calculated to identify the strongest association between PROs and satisfaction. This guided the following decision to build four distinct patient satisfaction groups. These clusters were based on two determinants, i.e. (i) satisfaction levels and (ii) whether or not reaching minimal clinically important differences (MCID) [29]. Next, we performed explorative analyses using chi-squared tests and analysis of variance (ANOVA) to understand the basic associations between explanatory variables and patient clusters (see below). Finally, we used results of the explorative analysis to perform multinomial logistic regression models to examine the relationship between patient clusters and potential predictors and to understand the effect of each predictor on the likelihood of being in the different patient satisfaction clusters.

#### Patient satisfaction groups

Both the 12-months PRO and the PRO change were correlated to the 12-month post-surgery satisfaction levels. The PROM indicator with the highest correlation coefficient was selected for the next analysis step (i.e. building the clusters), hypothesizing that the highest correlation represents the PROM with the largest impact on the level of satisfaction. The satisfaction levels were dichotomized to “satisfied” (combining satisfaction levels “very satisfied” and “satisfied”) and “dissatisfied” (combining satisfaction levels “neither satisfied nor unsatisfied”, “unsatisfied” and “very unsatisfied”).

**Fig. 2:**
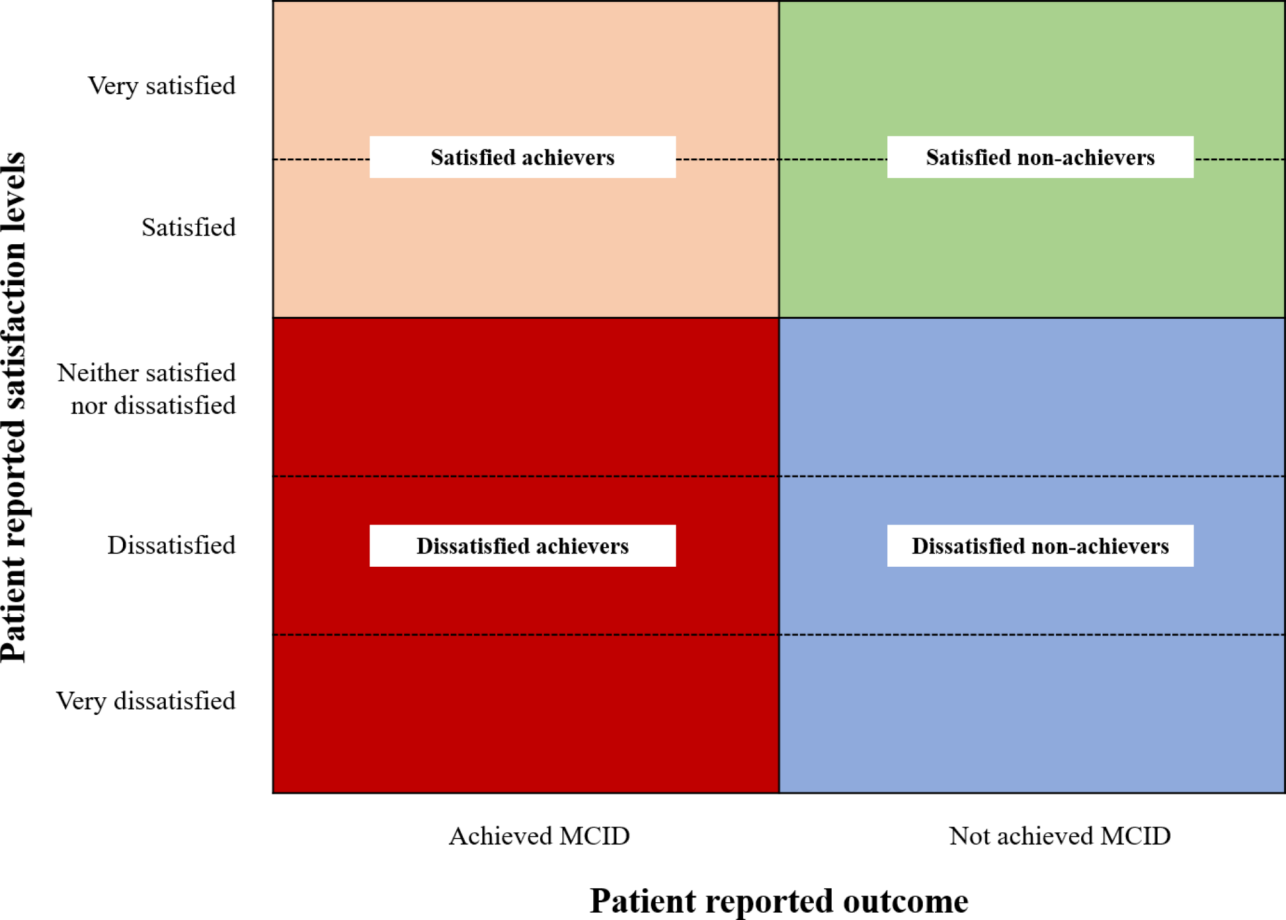
Patient groups based on patient-reported outcome and satisfaction

Based on the preceding correlation analysis we built four distinct patient groups (see Fig. 2):

- Group A – Satisfied Achievers: Patients were satisfied and their PRO change between hospital admission and 12-month follow-up reached the MCID.
- Group B – Dissatisfied Achievers: Patients were not satisfied but their PRO change between hospital admission and 12-month follow-up reached the MCID.
- Group C – Satisfied Non-Achievers: Patients were satisfied but their PRO change between hospital admission and 12-month follow-up did not reach the MCID.
- Group D – Dissatisfied Non-Achievers: Patients were not satisfied and their PRO change between hospital admission and 12-month follow-up did not reach the MCID.

#### Explorative analyses

We first performed an explorative analysis to explore patterns and associations in the data. This aimed to identify potential predictors for group assignment and determine relevant variables worth including in the regression model.

Chi-squared tests were used to identify significant associations between categorical variables and the four patient groups. Categorical variables included in the chi-squared analysis were gender, living situation, education, hospital of treatment, mobilization, and different patient comorbidities. To further investigate the significant associations identified by the chi-squared tests, we conducted bonferroni-corrected post hoc analyses to control for type I errors in multiple comparisons. Due to the data safety regulations, the exact patient numbers per hospital cannot be shown in the included tables.

Further, ANOVA was performed to identify significant associations between continuous variables and the four patient groups. Continuous variables included in the ANOVAs were age, admission PROs, 12-month PROs and PRO change. The results of this explorative analysis guided the selection of variables for the multinomial logistic regression, ensuring that only relevant predictors were included. Following the ANOVAs, we utilized Tukey’s Honestly Significant Difference (HSD) test to determine which specific group means differed significantly, ensuring a robust comparison across all pairs of groups.

#### Regression analysis

Based on the preceding results of the explorative analysis we built a multinomial logistic regression to model the relationship between the multi-category dependent variable (i.e. the patient groups) and the potential predictors. We quantified the strength and direction of association as relative risk ratio (RRR) of each predictor. RRRs reflect the probability of an outcome occurring in one group over the probability of its occurrence in a reference group. The reference group was Group A – Satisfied Achievers. An RRR of 1 means that the risk is the same in both groups, greater than 1 means a higher risk in the first group (compared to the reference), and less than 1 means a lower risk. The multinomial logistic regression model enabled conclusions on the likelihood of a patient falling into one of the four groups based on their characteristics while controlling for potential confounding variables. Variables included in the regression model were age, gender, BMI, comorbidities, education and mobilization after surgery (rapid recovery < 6 h after surgery vs. conventional care). Three different models were run including the previously mentioned variables: (1) the admission PROs (PROMIS-fatigue, PROMIS-depression, pain, EQ-5D-5 L and EQ-VAS) (2) the 12-months PROs and (3) the PRO change.

Missing data was imputed using the MissForest package in R. All analyses were run with STATA version 13.1.

## Results

### Patient groups

We examined the pearson correlation coefficient between all PROs and the patient satisfaction levels to identify the strongest association. Based on this we intended to define the distinct patient groups. We found that for both, the 12-month PROs and the PRO change, the KOOS-PS showed the strongest association with patient satisfaction (12-month PRO *r* = 0.6; PRO change *r* = 0.45). Therefore, in the following steps we built patient groups according to the framework shown in Fig. 2 based on whether patients were satisfied (yes/no) and an MCID in KOOS-PS was achieved (yes/no). Since MCIDs are only available for longitudinal comparisons, the PRO change was used to define the patient clusters. Concludingly, KOOS-PS was excluded from the explorative and regression analyses.

### Descriptive statistics of the patient groups

Among the 1546 knee replacement patients, most patients were identified as Satisfied Achievers (1146) as shown in Table 1 together with the descriptive statistics. The remaining three groups were smaller and had similar sizes ranging from 128 Dissatisfied Non-Achievers and 131 Dissatisfied Achievers to 141 Satisfied Non-Achievers. In total 54.1% were female patients, while only in the group of Satisfied Non-Achievers the share of male patients was higher (54.6% male patients). The average age of the population was 66 years, with the Dissatisfied Non-Achievers being the youngest group with 64 years as the average age and the Satisfied Non-Achievers the oldest group (68 years on average). The Dissatisfied Non-Achiever group had the highest average BMI (31.22). Both dissatisfied groups (Dissatisfied Achievers and Dissatisfied Non-Achievers) had the highest share of back comorbidities (49.62 and 42.19 respectively) and various other comorbidities (depression, heart-related diseases, circulation-related diseases and neurological diseases).

**Table 1:** Descriptive statistics of the study population

	Group A: Satisfied achievers			Group B: Dissatisfied achievers			Group C: Satisfied non-achievers			Group D: Dissatisfied non-achievers		
	female	male	Total	female	male	Total	female	male	Total	female	male	Total
**N (%)**	**625** **(54.54)**	**521** **(45.46)**	**1146** **(74**,**13**)	**78** **(59.54)**	**53** **(40.46)**	**131** **(8.47)**	**64 (45.39)**	**77** **(54.61)**	**141** **(9.12)**	**69** **(53.91)**	**59** **(46.09)**	**128** **(8.28)**
**Age**,** mean (SD)**	66.25 (9.33)	65.59 (9.32)	65.95 (9.33)	64.36 (9.54)	66.75 (9.36)	65.33 (9.50)	67.64 (9.59)	67.64 (9.39)	67.66 (9.45)	62.96 (8.38)	65.51 (9.47)	64.13 (9.18)
**BMI**,** mean (SD)**	30.65 (5.99)	29.94 (5.03)	30.32 (5.58)	31.1 (5.79)	31.39 (4.66)	31.22 (5.34)	31.12 (6.59)	30.02 (5.38)	30.52 (5.96)	30.71 (7.16)	29.73 (5.67)	30.26 (6.51)
**Living Situation**,** n (%)**												
I live alone	159 (25.44)	59 (11.32)	218 (19.02)	16 (20.51)	7 (13.21)	23 (17.56)	20 (31.25)	7 (9.09)	27 (19.15)	15 (21.74)	6 (10.17)	21 (16.41)
I live in a care facility	3 (0.48)	2 (0.38)	5 (0.44)	0 (0)	1 (1.89)	1 (0.76)	0 (0.00)	0 (0.00)	0 (0.00)	1 (1.45)	2 (3.39)	3 (2.34)
I live with a partner/ family/friends	457 (73.12)	458 (87.91)	915 (79.84)	59 (75.64)	45 (84.91)	104 (79.39)	44 (68.75)	69 (89.61)	113 (80.14)	51 (73.91)	50 (84.75)	101 (78.91)
other	6 (0.96)	2 (0.38)	8 (0.70)	3 (3.85)	0 (0.00)	3 (2.29)	0 (0)	1 (1.30)	1 (0.71)	2 (2.9)	1 (1.69)	3 (2.34)
**Education**,** n (%)**												
high/middle school degree	414 (66.24)	273 (52.4)	687 (59.95)	50 (64.1)	35 (66.04)	85 (64.89)	42 (65.63)	44 (57.14)	86 (60.99)	37 (53.62)	32 (54.24)	69 (53.91)
no school degree	0 (0)	3 (0.58)	3 (0.26)	1 (1.28)	0 (0)	1 (0.76)	0 (0)	0 (0)	0 (0)	1 (1.45)	0 (0)	1 (0.78)
primary school degree	100 (16)	95 (18.23)	195 (17.02)	18 (23.08)	9 (16.98)	27 (20.61)	10 (15.63)	16 (20.78)	26 (18.44)	13 (18.84)	10 (16.95)	23 (17.97)
university degree	111 (17.76)	150 (28.79)	261 (22.77)	9 (11.54)	9 (16.98)	18 (13.74)	12 (18.75)	17 (22.08)	29 (20.57)	18 (26.09)	17 (28.81)	35 (27.34)
**Comorbidity**,** n (%)**												
back	176 (28.16)	121 (23.22)	297 (25.92)	47 (60.26)	18 (33.96)	65 (49.62)	17 (26.56)	20 (25.97)	37 (26.24)	32 (46.38)	22 (37.29)	54 (42.19)
depression	65 (10.40)	22 (4.22)	87 (7.59)	17 (21.79)	0 (0.00)	17 (12.98)	4 (6.25)	1 (1.30)	5 (3.55)	13 (18.84)	7 (11.86)	20 (15.63)
diabetes	58 (9.28)	55 (10.56)	113 (9.86)	12 (15.38)	10 (18.87)	22 (16.79)	4 (6.25)	13 (16.88)	17 (12.06)	6 (8.70)	9 (15.25)	15 (11.72)
heart	61 (9.67)	89 (17.08)	150 (13.09)	11 (14.10)	15 (28.30)	26 (19.85)	9 (14.06)	13 (16.88)	22 (15.60)	7 (10.14)	19 (32.20)	26 (20.31)
arthritis	67 (10.72)	37 (7.10)	104 (9.08)	14 (17.95)	8 (15.09)	22 (16.79)	10 (15.63)	8 (10.39)	18 (12.77)	13 (18.84)	6 (10.17)	19 (14.84)
cancer	40 (6.40)	29 (5.57)	69 (6.02)	6 (7.69)	4 (7.55)	10 (7.63)	4 (6.25)	3 (3.90)	7 (4.96)	1 (1.45)	5 (8.47)	6 (4.69)
stroke	15 (2.40)	17 (3.26)	32 (2.79)	6 (7.69)	1 (1.89)	7 (5.34)	1 (1.56)	2 (2.60)	3 (2.13)	3 (4.35)	4 (6.78)	7 (5.47)
blood	367 (58.72)	314 (60.27)	681 (59.42)	47 (60.25)	39 (73.58)	86 (65.65)	36 (56.25)	49 (63.64)	85 (60.28)	42 (60.87)	37 (62.71)	79 (61.72)
circulation	34 (5.44)	32 (6.14)	66 (5.76)	12 (15.39)	9 (16.98)	21 (16.03)	6 (9.38)	5 (6.49)	11 (7.80)	9 (13.04)	11 (18.64)	20 (15.63)
lung	93 (14.88)	48 (9.21)	141 (12.30)	14 (17.95)	5 (9.43)	19 (14.50)	4 (6.25)	13 (16.88)	17 (12.06)	10 (14.49)	5 (8.47)	15 (11.72)
neuro	20 (3.20)	11 (2.11)	31 (2.72)	0 (0.00)	2 (3.77)	2 (1.53)	4 (6.25)	1 (1.30)	5 (3.55)	9 (13.04)	1 (1.69)	10 (7.81)
**Admission PRO**,** mean (SD)**^**a**^												
EQ-VAS (HrQoL)	56.69 (18.33)	60.72 (19.23)	58.52 (18.85)	46.5 (19.12)	58.02 (19.78)	51.16 (20.13)	61.27 (18.99)	63.4 (19.98)	62.43 (19.5)	51.32 (17.87)	58.32 (18.16)	54.55 (18.27)
EQ-5D-5 L (HrQoL)	0.60 (0.24)	0.66 (0.23)	0.63 (0.24)	0.48 (0.29)	0.59 (0.25)	0.52 (0.28)	0.71 (0.22)	0.75 (0.20)	0.73 (0.21)	0.53 (0.27)	0.65 (0.23)	0.58 (0.26)
PROMIS-depression	50.56 (8.06)	47.15 (7.67)	49.01 (8.07)	54.89 (7.71)	51.18 (8.02)	53.39 (8.01)	48.84 (7.59)	45.2 (6.07)	46.85 (7.02)	53.85 (8.14)	48.61 (8.26)	51.43 (8.58)
PROMIS-fatigue	49.4 (9.54)	46.33 (9.44)	48 (9.61)	53.27 (9.06)	48.79 (9.42)	51.46 (9.43)	46.33 (7.87)	42.74 (7.62)	44.37 (7.91)	51.8 (8.42)	48.28 (9.60)	50.18 (9.12)
KOOS-PS (physical functioning)	44.89 (11.4)	41.57 (10.8)	43.38 (11.24)	53.31 (14.12)	50.07 (15.08)	52 (14.55)	34.2 (9.13)	32.61 (11.78)	33.33 (10.65)	43.16 (8.13)	38.12 (9.66)	40.84 (9.19)
Pain	2.77 (1.26)	2.50 (1.18)	2.65 (1.23)	3.25 (1.42)	2.89 (1.57)	3.11 (1.49)	2.61 (1.31)	2.54 (1.57)	2.57 (1.45)	2.99 (1.28)	2.63 (1.15)	2.83 (1.23)
**Fast-track mobilisation**,** n (%)**	276 (44.16)	242 (46.45)	518 (45.20)	35 (44.87)	22 (41.51)	57 (43.51)	39 (60.94)	38 (49.35)	77 (54.61)	29 (42.03)	28 (47.46)	57 (44.53)
**Readmissions**	6 (0.96)	14 (2.69)	20 (1.75)	7 (8.97)	3 (5.66)	10 (7.63)	1 (1.56)	2 (2.60)	3 (2.13)	6 (8.90)	1 (1.69)	7 (5.47)
**Reoperations**	12 (0.02)	10 (1.92)	22 (1.92)	5 (6.41)	2 (3.77)	7 (5.34)	4 (6.25)	3 (3.90)	6 (4.26)	6 (8.90)	2 (3.39)	7 (5.47)

Group A: Diff. 12-month KOOS – Pre-OP-KOOS > MCID & Satisfied

Group B: Diff. 12-month KOOS – Pre-OP-KOOS > MCID & Dissatisfied

Group C: Diff. 12-month KOOS – Pre-OP-KOOS < MCID & Satisfied

Group D: Diff. 12-month KOOS – Pre-OP-KOOS < MCID & Dissatisfied

^a^ Patient-reported outcome measure (PROM)-scores have different score ranges: -0.661-1 for the EQ-5D-5 L, 0-100 for the EQ-VAS, 0-100 for the HOOS-PS and KOOS-PS, 33.7–75.8 for the PROMIS-fatigue and 41-79.4 for the PROMIS-depression. Higher values in the EQ-VAS and EQ-5D-5 L indicate better health levels, whereas lower values in H/KOOS-PS, PROMIS-fatigue, and depression indicate better health (less health impairment)

### Explorative analyses

We investigated the association between group allocation and various categorical patient, treatment, comorbidity, and provider characteristics using chi-square (χ²) tests and Cramer’s V to assess the effect size of these associations. The results are summarized in Table 2. Among the patient characteristics, none showed a statistically significant association with group allocation (*p* > 0.05).

**Table 2:** Chi-Square test results of the patient, provider and treatment characteristics and comorbidities the group allocation

	Variable	χ²	*p*-value	V
Patient characteristics	Gender	5.96	0.114	0.06
	Smoker	5.20	0.157	0.06
	Education	10.59	0.304	0.05
	Living Situation	14.49	0.106	0.06
Treatment characteristics	Mobilization	4.93	0.177	0.06
	Readmission	20.79***	0.000	0.12
	Reoperation	11.11**	0.011	0.08
Comorbidities	Back	43.34***	0.000	0.17
	Depression	17.64***	0.001	0.11
	Cancer	1.28	0.733	0.03
	Stroke	5.25	0.154	0.06
	Blood	2.03	0.565	0.03
	Diabetes	6.27*	0.099	0.06
	Circulation	30.41***	0.000	0.14
	Neuro	11.59***	0.009	0.09
	Heart	8.49**	0.037	0.07
	Arthritis	11.29**	0.010	0.09
Provider characteristics	Hospital	39.29**	0.025	0.09

Table 2 Shows the results of the chi-squared tests to examine the association between group allocation and categorical variables, i.e. patient characteristics, treatment characteristics and pre-diagnosed comorbidities. The effect size is given as Cramer’s V (V). Significance markers: *p < 0.1 **p < 0.05 ***p < 0.01

Among treatment characteristics, significant associations were found for readmission (χ² = 20.79, *p* < 0.01, V = 0.12) and reoperation (χ² = 11.11, *p* < 0.05, V = 0.08). Mobilization did not show a significant association.

Several comorbidities were significantly associated with group allocation. These included back problems (χ² = 43.34, *p* < 0.01, V = 0.17), depression (χ² = 17.64, *p* < 0.01, V = 0.11), blood circulation issues (χ² = 30.41, *p* < 0.01, V = 0.14), neurological conditions (χ² = 11.59, *p* < 0.01, V = 0.09), heart disease (χ² = 8.49, *p* < 0.05, V = 0.07), and arthritis (χ² = 11.29, *p* < 0.05, V = 0.09). Other comorbidities such as cancer, stroke, blood disorders, and diabetes did not show significant associations.

Finally, the treating hospital was significantly associated with group allocation (χ² = 39.29, *p* < 0.05, V = 0.09).

These findings indicated that several treatment characteristics and comorbidities, as well as provider characteristics, were significantly associated with group allocation, with varying degrees of effect size as indicated by Cramer’s V. However, even though we found these significant associations, following Ellis [30], effect sizes between 0.1 and 0.3 are considered only small effects.

The results of the ANOVAs, including F-statistic, *p*-values, and effect sizes (η²), are summarized in Table 3.

**Table 3:** ANOVA results for all PROMs per measurement time

Event	Variable	F	*p*	η²
Admission PRO	EQ-5D-5 L	18.9	0.000	0.04
	EQ-VAS	9.94	0.000	0.02
	PROMIS-D-SF	19.23	0.000	0.04
	PROMIS-F-SF	15.05	0.000	0.03
	Pain	5.87	0.001	0.01
12-month PRO	EQ-5D-5 L	201.46	0.000	0.28
	EQ-VAS	121.66	0.000	0.19
	PROMIS-D-SF	81.61	0.000	0.14
	PROMIS-F-SF	93.21	0.000	0.15
	Pain	161.91	0.000	0.24
PRO-change	EQ-5D-5 L	86.82	0.000	0.14
	EQ-VAS	58.48	0.000	0.10
	PROMIS-D-SF	43.64	0.000	0.08
	PROMIS-F-SF	68.84	0.000	0.12
	Pain	99.34	0.000	0.16

Table 3 shows the results of the ANOVAs to examine the association between group allocation and numerical variables, i.e. patient-reported outcomes. The effect size is given as eta-squared (η²). EQ-5D-5 L = EuroQol five dimensions five levels questionnaire, EQ-VAS = EuroQol visual analogue scale, PROMIS-D-SF = Patient Reported Outcomes Measurement Information System (PROMIS) depression shortform, PROMIS-F-SF = Patient Reported Outcomes Measurement Information System fatigue shortform, KOOS-PS = Knee injury and Osteoarthritis Outcome Score Physical Function Shortform

Overall, we found that all PROs at both measurement times as well as the change over time were significantly associated with the group allocation, whereas 12-month PROs and the PRO change showed larger effect sizes compared to the baseline scores. According to Cohen [31], the effect size thresholds are 0.01 (small effect), 0.06 (medium effect), and 0.14 (large effect). Based on these thresholds, our findings indicated that several PROs, particularly KOOS-PS, EQ-5D-5 L, and pain, showed substantial differences among the groups, especially at the 12-month follow-up and in the change scores while the effect sizes for the baseline values were smaller.

At baseline, significant differences among the groups were observed for all admission PROs. The effect sizes indicated small to moderate effects for EQ-5D-5 L (F = 18.9, *p* < 0.001, η² = 0.04), EQ-VAS (F = 9.94, *p* < 0.001, η² = 0.02), PROMIS-D-SF (F = 19.23, *p* < 0.001, η² = 0.04), and PROMIS-F-SF (F = 15.05, *p* < 0.001, η² = 0.03). The KOOS-PS demonstrated a moderate effect (F = 63.43, *p* < 0.001, η² = 0.11), while pain showed a small effect (F = 5.87, *p* = 0.001, η² = 0.01).

All 12 months PROs showed highly significant differences among the groups with large effect sizes. The EQ-5D-5 L had the largest effect size (F = 201.46, *p* < 0.001, η² = 0.28), followed by pain (F = 161.91, *p* < 0.001, η² = 0.24), EQ-VAS (F = 121.66, *p* < 0.001, η² = 0.19), PROMIS-F-SF (F = 93.21, *p* < 0.001, η² = 0.15), and PROMIS-D-SF (F = 81.61, *p* < 0.001, η² = 0.14).

For the PRO-change, significant differences among the groups were observed for all PROs, with effect sizes ranging from moderate to large. Pain showed the largest effect size (F = 99.34, *p* < 0.001, η² = 0.16), followed by EQ-5D-5 L (F = 86.82, *p* < 0.001, η² = 0.14), PROMIS-F-SF (F = 68.84, *p* < 0.001, η² = 0.12), EQ-VAS (F = 58.48, *p* < 0.001, η² = 0.10), and PROMIS-D-SF (F = 43.64, *p* < 0.001, η² = 0.08).

### Regression analyses

**Fig. 3:**
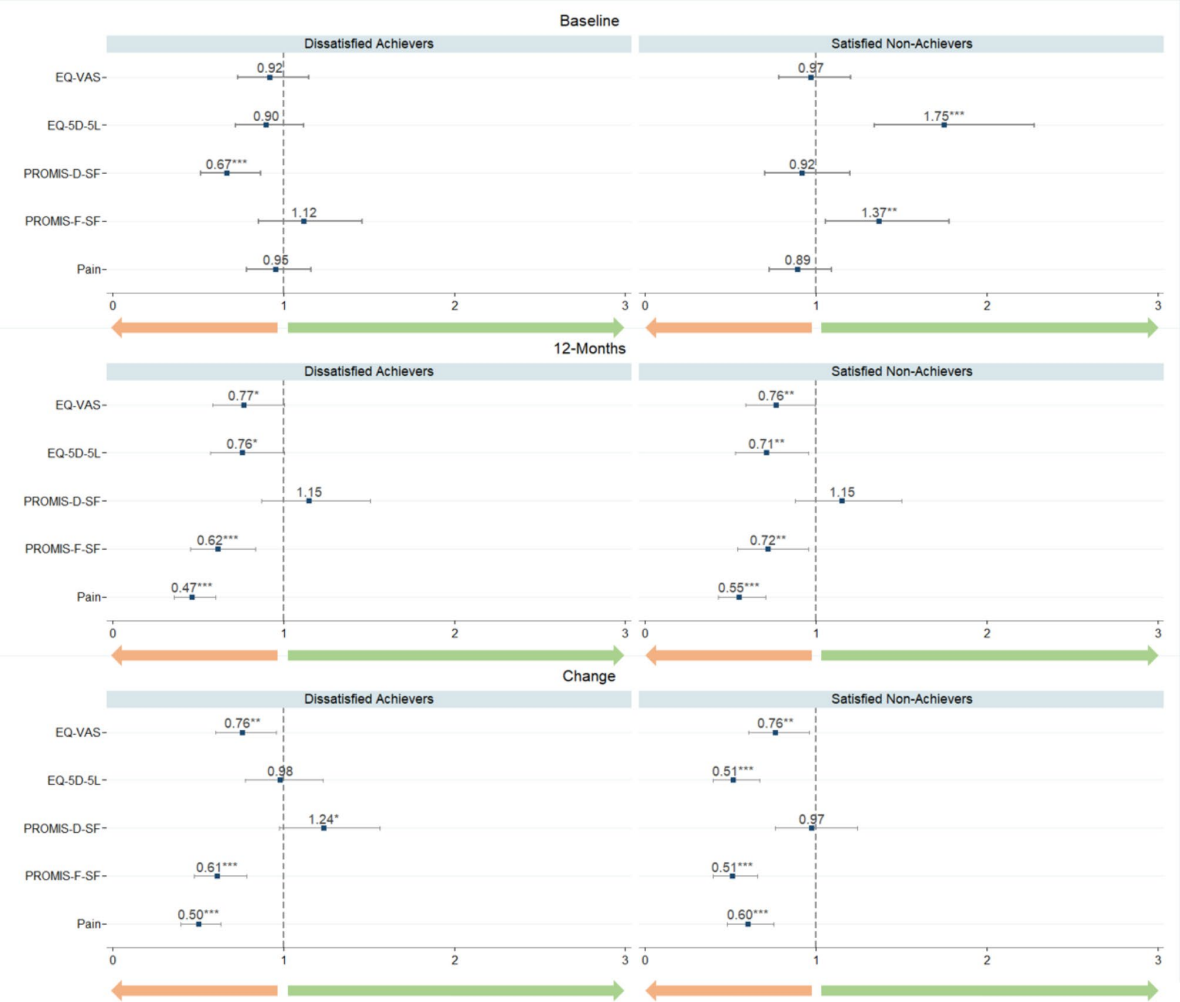
Dot-whisker plot of the multinomial logit regression in relative risk ratios

In Fig. 3, the results of the PRO-related coefficients are displayed based on three different models (admission PRO values, 12-month PRO values and PRO-change values), while controlling for other patient and treatment characteristics. The baseline model examines the association between group allocation and admission PROs, the 12-month model between group allocation and 12-month PROs, and the change model between group allocation and the PRO change between baseline and 12-month post-surgery. Tables 1, 2 and 3 in the Appendix present the full results of the multinomial logistic regression models. Due to a lack of patients who reported a kidney or liver-related comorbidity or were readmitted to the hospital, these variables were excluded from the regression model.

The first panel of Fig. 3 shows that lower depression scores (PROMIS-D-SF) at baseline were significantly associated with a decreased relative risk of being a Dissatisfied Achiever (0.67; 95% CI [0.52, 0.87], *p* = 0.002). At the same time, higher (better) baseline scores in EQ-5D-5 L and PROMIS-F-SF led to an increased probability of being a Satisfied Non-Achiever (EQ-5D-5 L: 1.75; 95% CI [1.34, 2.28]; *p* < 0.001; PROMIS-F-SF: 1.37; 95% CI [1.05, 1.78]; *p* = 0.018). The remaining baseline PROM scores did not show a significant association for the group allocation.

Next, the middle panel of Fig. 3 shows that, with the exception of PROMIS-D-SF, all remaining 12-month PROs were at least weakly associated with the group allocation. Increased (better) 12-month scores in the HRQoL PROMs EQ-VAS and EQ-5D-5 L led to a weak significant decrease in the relative risk to be a Dissatisfied Achiever (EQ-VAS: 0.77; 95% CI [0.58; 1.00]; *p* = 0.054; EQ-5D-5 L: 0.76; 95% CI [0.57; 1.01]; *p* = 0.055) and to significantly decreased likelihood to be a Satisfied Non-Achiever (EQ-VAS: 0.76; 95% CI [0.59; 1.00]; *p* = 0.046; EQ-5D-5 L: 0.71; 95% CI [0.53; 0.96]; *p* = 0.025). Increased (better) 12-month scores in PROMIS-F-SF were associated with a highly significant decrease in the likelihood of being in the cluster of Dissatisfied Achievers (0.62; 95% CI [0.45; 0.84]; *p* = 0.002) and a significant decrease in the likelihood of being a Satisfied Non-Achiever (0.72; 95% CI [0.54; 0.96]; *p* = 0.023). Finally, the 12-month pain level showed the strongest association with the group allocation. The lower the pain level at 12 months, the smaller the relative risk of being a Dissatisfied Achiever (0.47; 95% CI [0.36; 0.60]; *p* < 0.001) or Satisfied Non-Achiever (0.55; 95% CI [0.43; 0.70]; *p* < 0.001). In other words, the more pain at 12 months post-surgery, the more likely patients were to be Dissatisfied Achievers or Satisfied Non-Achievers.

The last panel of Fig. 3 shows that when MCIDs were achieved, particularly higher improvements in EQ-VAS (0.76; 95% CI [0.60; 0.96]; *p* = 0.021), in PROMIS-F-SF (0.61; 95% CI [0.48; 0.79]; *p* < 0.001), and pain (0.50; 95% CI [0.40; 0.63]; *p* < 0.001) a significantly decreased likelihood of being Dissatisfied Achievers was observed. At the same time, higher improvements in EQ-VAS (0.76; 95% CI [0.60; 0.96]; *p* = 0.022), EQ-5D-5 L (0.51; 95% CI [0.40; 0.67]; *p* < 0.001), PROMIS-F-SF (0.51; 95% CI [0.40; 0.66]; *p* < 0.001), and pain (0.60; 95% CI [0.48; 0.75]; *p* < 0.001) significantly decreased the likelihood of being a Satisfied Non-Achiever. From all three models, the last model, PRO change, had the highest explanatory power with R²=0.24.

Figure 3 displays the relative risk ratios (RRR) resulting from the multinomial logit regression. The grey lines represent the corresponding 95% confidence intervals. The RRR indicate the probability of a group allocation under one unit increase of the corresponding dependent variable relative to the reference category “Satisfied Achievers”. RRR greater than 1 indicates increased relative risk, while RRR smaller than 1 indicate decreased relative risk. The upper panel shows the RRR under consideration of baseline PROMs, the middle panel of 12-month post-surgery PROMs, and the bottom panel of the change in PROM scores between baseline and 12-month post-surgery. PROMs = Patient-Reported Outcome Measures, green indicates higher RRR with better health outcomes measured through PROMs and orange indicates lower RRR with better health outcomes

#### Comorbidities

Further, as seen in Tables 1, 2 and 3 in the Appendix, various comorbidities showed a significant association with the patient group assignment. For instance, a registered comorbidity of blood circulation, chronic back pain or diabetes increased the likelihood of being a Dissatisfied Achiever. Pre-diagnosed depression, on the other hand, decreased the likelihood of being a Satisfied Non-Achiever.

## Discussion

In this total knee replacement study population, most patients (74%) were identified as Satisfied Achievers, experiencing a clinically meaningful improvement in knee functionality and reporting to be satisfied with the results of surgery. The other three groups were smaller with 8% Dissatisfied Non-Achievers and Dissatisfied Achievers respectively and 9% Satisfied Non-Achievers. In the exploratory analyses, we found that all other PROs (pain, fatigue, depression and HrQoL) were associated with the group allocation with a small to moderate effect size at all measurement times. 12-month PROs and the PRO change showed larger effect sizes compared to the admission PROs. In addition, some treatment characteristics were associated with the group allocation with a small effect size (readmission and reoperation) and comorbidities of patients with a moderate effect size (back problems, depression, blood circulation issues, neurological conditions, heart disease and arthritis). Various reasons could have led to the fact, that the hospital was also significantly associated with the group allocation. Those include differences in the served patient population, which could not be accounted for in the analyses, or differences in processes or structures in each of the hospitals, which improve or worsen satisfaction or health outcomes.

In the main analyses, the multinomial logistic regressions controlled for other confounding variables, confirming most of the findings from the exploratory analyses. We confirmed that there was a higher relative risk of belonging to the Dissatisfied Achiever group when experiencing other health-related symptoms including the PROs in other health dimensions and specific comorbidities. Higher HrQoL, fatigue and pain improvements were negatively associated with this group affiliation while having back pain and circulatory comorbidities increased the likelihood of being a Dissatisfied Achiever. These findings indicate that other health-related sources of dissatisfaction are present. These insights could be used in preoperative patient education programs or to modulate patients’ expectations during the shared decision process, thus possibly positively impacting satisfaction. There is some evidence that patient education is effective in reducing pain and improving function in patients with knee osteoarthritis [32]. Moreover, pre- and peri-operative education to manage expectations has been shown to increase satisfaction among knee replacement patients [33, 34].

As satisfaction is frequently used in benchmarking, our findings can inform the data collection and selection of risk adjustment variables such as back pain and circulatory diseases.

Patients that were classified as Satisfied Non-Achievers were at higher relative risk of lower PRO-changes in other health dimensions, specifically in HrQoL, fatigue and pain as well as higher (better) admission scores in the EQ-5D-5L and fatigue. One reason could be the correlation between positive changes in physical function (KOOS-PS) and the other PROs. This could be because other health dimensions like HRQoL often do not improve, if physical function does not improve. Moreover, as previously discussed in the orthopedic literature, the PRO at admission has a high predictive performance on the PRO change experienced by patients and can even be a reference point to determine who will likely benefit from surgery [35]. Hence, the Satisfied Non-Achievers could be Non-Achievers, as it becomes less likely to achieve a meaningful improvement with already very good admission scores. This puts into question (1) the appropriateness of surgery for this group of patients and (2) the meaningfulness of measuring patients’ satisfaction to assess surgery quality. Patients might be satisfied as they feel cared for, which does not necessarily coincide with a meaningful improvement in knee function. There is also the possibility that this group of patients would benefit more from non-operative treatment options, like physical therapy and physiotherapy treatment modalities. Hence, the question arises, whether a closer evaluation of physical function levels during the shared decision process would help to decide whether surgery (TKA) would be the best option for this patient group. In a qualitative study on the impact of patient factors on the decision to progress to TKA, it was found that missed opportunities in general practice to recommend patients to first try non-surgical interventions were highlighted by the patients as influencing factors to decide for TKA [36].

This study comes with some limitations. The analyses presented in this paper focus on the relationship between patient outcomes and patient satisfaction within a 12-month follow-up period, as determined by the PROMoting Quality study design. While TKA is a high-volume procedure associated with relatively high and rapid recovery expectations, the question remains on how the observed relationships between PROs and patient satisfaction might evolve when exceeding the follow-up period of 12 months, and how it evolves leading up to the measurement 12-months post-surgery. Other studies on TKA outcomes suggest follow-up periods of up to 5 years [37]. Accordingly, it remains unclear whether the present results will remain robust over a longer period. Furthermore, we were only able to examine the potential predictors that were collected as part of the PROMoting Quality study. There are other important factors that could have influenced patient satisfaction, such as the surgical technique, implant design or the component fixation method (cementless, cemented or hybrid) used during surgery [38, 39]. The choice of different surgical techniques or component fixation methods are already shown to influence patient outcomes [40–43]. Therefore, they are likely to influence satisfaction, too, and introduce variability in the results. Lastly, besides the above described included potential predictors, other more rigorous evaluations of preoperative physical impairment could not be included, since this was not accessed within the PROMoting Quality study. Assessments of physical function based on functional tasks, like the timed-up and go test, could have helped to cluster the patients based on a functional task, instead of a score. Furthermore, preoperative radiological data on the grade of cartilage damage and the presence and severity of patellofemoral joint cartilage disease could have been used as a potential predictor too, since there is evidence, that these have an impact on functional outcomes after TKA [44, 45]. Future studies should build upon these limitations to generate further insights into the relationship between health outcomes and satisfaction in patients scheduled for TKA.

## Conclusion

74% of patients in the study population were Satisfied Achievers, the best-case scenario after TKA. Around 17% of the patients were allocated to one of the two groups that this study aimed to understand better, the Dissatisfied Achievers and the Satisfied Non-Achievers. Other health-related sources of dissatisfaction increased the relative risk of being a Dissatisfied Achiever. These included pain, HrQoL, fatigue, back pain and circulatory comorbidities. Addressing these health concerns e.g. through expectation management and weighing up alternative or supplementary treatment options could be a way to improve satisfaction. Preoperative education could include a more detailed discussion of potential comorbidities and their impact on outcomes, as well as the establishment of realistic recovery expectations. Patients presenting with significant comorbid conditions, such as chronic pain or fatigue, might benefit from referral to multidisciplinary prehabilitation to optimize their health status before surgery. Conservative treatment options like physical therapy or physiotherapy and a closer evaluation at which physical impairment level surgery is beneficial could help to improve the care of Satisfied Non-Achievers.

## Electronic supplementary material

Below is the link to the electronic supplementary material.

Supplementary Material 1

## Data Availability

Since the data contains sensitive patient information it is legally not allowed to make the data publicly accessible to others due to the German data protection law and the data protection agreements within the trial. In order to enable verifiability of the study results after completion of the project (09/2023), the data will be stored at the PROMoting Quality research institutions - TU Berlin and aQua Institute - for a period of 10 years after project completion. Data access can only be granted in exceptional cases.

## References

[1] D’Ambrosi R, Ursino C, Mariani I, Ursino N, Formica M, Chen AF. Clinical outcomes, complications, and survivorship for unicompartmental knee arthroplasty versus total knee arthroplasty in patients aged 80 years and older with isolated medial knee osteoarthritis: a matched cohort analysis. Arch Orthop Trauma Surg. 2023;143(10):6371–9. 10.1007/s00402-023-04916-9 37244888 10.1007/s00402-023-04916-9 PMC10491502

[2] Puvanendran A, Jaibaji M, Volpin A, Konan S. Survivorship, clinical outcomes and indications for revision in uncemented unicompartmental knee arthroplasty: systematic review. Acta Orthop Belg. 2023;89(1):83–95. 10.52628/89.1.9873 37294990 10.52628/89.1.9873

[3] Cöster MC, Bremander A, Nilsdotter A. Patient-reported outcome for 17,648 patients in 5 different Swedish orthopaedic quality registers before and 1 year after surgery: an observational study. ActaO. 2023;94:1–7. 10.2340/17453674.2023.6577 10.2340/17453674.2023.6577 PMC9880767 36701121

[4] DeFrance MJ, Scuderi GR. Are 20% of patients actually dissatisfied following total knee arthroplasty? A systematic review of the literature. J Arthroplast. 2023;38(3):594–9. 10.1016/j.arth.2022.10.011 10.1016/j.arth.2022.10.011 36252743

[5] Ayers DC, Yousef M, Zheng H, Yang W, Franklin PD. The prevalence and predictors of patient dissatisfaction 5-years following primary total knee arthroplasty. J Arthroplast. 2022;37(6):S121–8. 10.1016/j.arth.2022.02.077 10.1016/j.arth.2022.02.077 35227816

[6] Clement ND, Bardgett M, Weir D, Holland J, Gerrand C, Deehan DJ. Three groups of dissatisfied patients exist after total knee arthroplasty: early, persistent, and late. Bone Joint J. 2018;100–B(2):161–9. 10.1302/0301-620X.100B2.BJJ-2017-1016.R1 29437057 10.1302/0301-620X.100B2.BJJ-2017-1016.R1

[7] Naal FD, Impellizzeri FM, Lenze U, Wellauer V, Von Eisenhart-Rothe R, Leunig M. Clinical improvement and satisfaction after total joint replacement: a prospective 12-month evaluation on the patients’ perspective. Qual Life Res. 2015;24(12):2917–25. 10.1007/s11136-015-1042-3 26068733 10.1007/s11136-015-1042-3

[8] Farooq H, Deckard ER, Ziemba-Davis M, Madsen A, Meneghini RM. Predictors of patient satisfaction following primary total knee arthroplasty: results from a traditional statistical model and a machine learning algorithm. J Arthroplast. 2020;35(11):3123–30. 10.1016/j.arth.2020.05.077 10.1016/j.arth.2020.05.077 32595003

[9] Ingelsrud LH, Wilkinson JM, Overgaard S, et al. How do patient-reported outcome scores in international hip and knee arthroplasty registries compare? Clin Orthop Relat Res. 2022;480(10):1884–96. 10.1097/CORR.0000000000002306 35901444 10.1097/CORR.0000000000002306 PMC9473760

[10] NHS England. Patient Reported Outcome Measures (PROMs). July 25. 2024. https://digital.nhs.uk/data-and-information/data-tools-and-services/data-services/patient-reported-outcome-measures-proms

[11] Lin E, Uhler LM, Finley EP, et al. Incorporating patient-reported outcomes into shared decision-making in the management of patients with osteoarthritis of the knee: a hybrid effectiveness-implementation study protocol. BMJ Open. 2022;12(2):e055933. 10.1136/bmjopen-2021-055933 35190439 10.1136/bmjopen-2021-055933 PMC8860037

[12] Pasqualini I, Piuzzi NS. New CMS Policy on the Mandatory Collection of patient-reported outcome measures for total hip and knee arthroplasty by 2027: what Orthopaedic surgeons should know. J Bone Joint Surg. 2024;106(13):1233–41. 10.2106/JBJS.23.01013 38335264 10.2106/JBJS.23.01013

[13] Catena R, Kirkegaard K, Nayak D, Tomini SM. A Novel Value-based procurement agreement to improve outcomes for patients undergoing knee replacement. NEJM Catalyst. 2024;5(6). 10.1056/CAT.23.0327

[14] Rolfson O, Wissig S, Van Maasakkers L, et al. Defining an International Standard Set of Outcome measures for patients with hip or knee osteoarthritis: Consensus of the International Consortium for Health Outcomes Measurement Hip and knee osteoarthritis Working Group. Arthritis Care Res. 2016;68(11):1631–9. 10.1002/acr.22868 10.1002/acr.22868 PMC5129496 26881821

[15] Kendir C, De Bienassis K, Slawomirski L et al. International Assessment of the Use and Results of Patient-Reported Outcome Measures for Hip and Knee Replacement Surgery: Findings of the OECD Patient-Reported Indicator Surveys (PaRIS) Working Group on Hip and Knee Replacement Surgery. Vol 148.; 2022. 10.1787/6da7f06b-en

[16] Black N, Varaganum M, Hutchings A. Relationship between patient reported experience (PREMs) and patient reported outcomes (PROMs) in elective surgery. BMJ Qual Saf. 2014;23(7):534–42. 10.1136/bmjqs-2013-002707 24508681 10.1136/bmjqs-2013-002707

[17] Abdelhameed MA, Abdelnasser MK, Zaky BR, Bakr HM, Aziz M, Mahran M. Preoperative stiffness is the most important predictor of postoperative patient’s satisfaction after total knee arthroplasty. Eur J Orthop Surg Traumatol. 2023;33(7):3019–24. 10.1007/s00590-023-03526-w 36947311 10.1007/s00590-023-03526-w PMC10504170

[18] Appiah KOB, Khunti K, Kelly BM, et al. Patient-rated satisfaction and improvement following hip and knee replacements: development of prediction models. Evaluation Clin Pract. 2023;29(2):300–11. 10.1111/jep.13767 10.1111/jep.13767 36172971

[19] Fan XY, Ma JH, Wu X, et al. How much improvement can satisfy patients? Exploring patients’ satisfaction 3 years after total knee arthroplasty. J Orthop Surg Res. 2021;16(1):389. 10.1186/s13018-021-02514-2 34140037 10.1186/s13018-021-02514-2 PMC8212506

[20] Klem NR, Smith A, O’Sullivan P, et al. What influences patient satisfaction after TKA? A qualitative investigation. Clin Orthop Relat Res. 2020;478(8):1850–66. 10.1097/CORR.0000000000001284 32732567 10.1097/CORR.0000000000001284 PMC7371044

[21] Steinbeck V, Langenberger B, Schöner L, et al. Electronic patient-reported outcome monitoring to improve quality of life after joint replacement: secondary analysis of a Randomized Clinical Trial. JAMA Netw Open. 2023;6(9):2331301. 10.1001/jamanetworkopen.2023.31301 10.1001/jamanetworkopen.2023.31301 PMC10474554 37656459

[22] Schöner L, Kuklinski D, Wittich L et al. Cost-effectiveness of a patient-reported outcome-based remote monitoring and alert intervention for early detection of critical recovery after joint replacement: A randomised controlled trial. Beard D, ed. PLoS Med. 2024;21(10):e1004459. 10.1371/journal.pmed.1004459 10.1371/journal.pmed.1004459 PMC11463742 39383175

[23] Kuklinski D, Oschmann L, Pross C, Busse R, Geissler A. The use of digitally collected patient-reported outcome measures for newly operated patients with total knee and hip replacements to improve post-treatment recovery: study protocol for a randomized controlled trial. Trials. 2020;21(1):322. 10.1186/s13063-020-04252-y 32272962 10.1186/s13063-020-04252-y PMC7147006

[24] Mathew G, Agha RSTROCSS. 2021: strengthening the reporting of cohort, cross-sectional and case-control studies in surgery. IJS Short Reports. 2021;6(4):e35-e35. 10.1097/SR9.0000000000000035 10.1016/j.ijsu.2021.106165 34774726

[25] Herdman M, Gudex C, Lloyd A, et al. Development and preliminary testing of the new five-level version of EQ-5D (EQ-5D-5L). Qual Life Res. 2011;20(10):1727–36. 10.1007/s11136-011-9903-x 21479777 10.1007/s11136-011-9903-x PMC3220807

[26] Perruccio AV, Stefan Lohmander L, Canizares M, et al. The development of a short measure of physical function for knee OA KOOS-Physical function shortform (KOOS-PS) – an OARSI/OMERACT initiative. Osteoarthr Cartil. 2008;16(5):542–50. 10.1016/j.joca.2007.12.014 10.1016/j.joca.2007.12.014 18294869

[27] PROMIS. User Manual and Scoring Instructions - PROMIS fatigue. Accessed October 24. 2024. https://www.healthmeasures.net/administrator/components/com_instruments/uploads/PROMIS Fatigue User Manual and Scoring Instructions_12July2024.pdf

[28] PROMIS. A brief guide to scoring PROMIS Depression instruments. Accessed October 24. 2024. https://www.healthmeasures.net/administrator/components/com_instruments/uploads/PROMIS Depression Scoring Manual_05Dec2023.pdf

[29] Langenberger B, Schrednitzki D, Halder AM, Busse R, Pross CM. Predicting whether patients will achieve minimal clinically important differences following hip or knee arthroplasty: a performance comparison of machine learning, logistic regression, and pre-surgery PROM scores using data from nine German hospitals. Bone Joint Res. 2023;12(9):512–21. 10.1302/2046-3758.129.BJR-2023-0070.R2 37652447 10.1302/2046-3758.129.BJR-2023-0070.R2 PMC10471446

[30] Ellis PD. The essential guide to Effect sizes: statistical power, Meta-analysis, and the Interpretation of Research Results. 1st ed. Cambridge University Press; 2010. 10.1017/CBO9780511761676

[31] Cohen J. Statistical Power Analysis for the behavioral sciences. 0 ed. Routledge; 2013. 10.4324/9780203771587

[32] Marques CJ, Bohlen K, Lampe F. Participation in a preoperative patient Education Session is a significant predictor of Better WOMAC Total Index Score and higher EQ-5D-5L Health Status Index 1 year after total knee and hip arthroplasties: a retrospective observational study. Am J Phys Med Rehabil. 2021;100(10):972–7. 10.1097/PHM.0000000000001689 33443861 10.1097/PHM.0000000000001689

[33] Tolk JJ, Janssen RPA, Haanstra TM, Van Der Steen MC, Bierma-Zeinstra SMA, Reijman M. The influence of expectation modification in knee arthroplasty on satisfaction of patients: a randomized controlled trial: the EKSPECT study. Bone Joint J. 2021;103–B(4):619–26. 10.1302/0301-620X.103B4.BJJ-2020-0629.R3 33789470 10.1302/0301-620X.103B4.BJJ-2020-0629.R3

[34] Zhao C, Liao Q, Yang D, Yang M, Xu P. Advances in perioperative pain management for total knee arthroplasty: a review of multimodal analgesic approaches. J Orthop Surg Res. 2024;19(1):843. 10.1186/s13018-024-05324-4 39696522 10.1186/s13018-024-05324-4 PMC11658298

[35] Langenberger B, Steinbeck V, Busse R. Who benefits from hip arthroplasty or knee arthroplasty? Preoperative patient-reported Outcome Thresholds Predict Meaningful Improvement. Clin Orthop Relat Res. 2024;482(5):867–81. 10.1097/CORR.0000000000002994 38393816 10.1097/CORR.0000000000002994 PMC11008644

[36] O’Brien P, Bunzli S, Ayton D, Dowsey MM, Gunn J, Manski-Nankervis JA. What are the patient factors that impact on decisions to progress to total knee replacement? A qualitative study involving patients with knee osteoarthritis. BMJ Open. 2019;9(9):e031310. 10.1136/bmjopen-2019-031310 31551388 10.1136/bmjopen-2019-031310 PMC6773346

[37] Ishii Y, Noguchi H, Sato J, Sakurai T, ichi Toyabe S. Quadriceps strength impairment in the mid- to long-term follow-up period after total knee arthroplasty. Knee Surg Sports Traumatol Arthrosc. 2017;25(11):3372–7. 10.1007/s00167-016-4333-5 27650527 10.1007/s00167-016-4333-5

[38] Sun K, Wu Y, Wu L, Shen B. Comparison of clinical outcomes among total knee arthroplasties using posterior-stabilized, cruciate-retaining, bi-cruciate substituting, bi-cruciate retaining designs: a systematic review and network meta-analysis. Chin Med J. 2023;136(15):1817–31. 10.1097/CM9.0000000000002183 37365688 10.1097/CM9.0000000000002183 PMC10406014

[39] Hantouly AT, Ahmed AF, Alzobi O, et al. Mobile-bearing versus fixed-bearing total knee arthroplasty: a meta-analysis of randomized controlled trials. Eur J Orthop Surg Traumatol. 2022;32(3):481–95. 10.1007/s00590-021-02999-x 34021791 10.1007/s00590-021-02999-x PMC8924090

[40] Bertin KC. Tibial component fixation in total knee arthroplasty. J Arthroplast. 2007;22(5):670–8. 10.1016/j.arth.2006.07.004 10.1016/j.arth.2006.07.004 17689774

[41] Xing P, Qu J, Feng S, Guo J, Huang T. Comparison of the efficacy of robot-assisted total knee arthroplasty in patients with knee osteoarthritis with varying severity deformity. J Orthop Surg Res. 2024;19(1):872. 10.1186/s13018-024-05372-w 39719605 10.1186/s13018-024-05372-w PMC11668079

[42] Bayoumi T, Burger JA, Van Der List JP, et al. Comparison of the early postoperative outcomes of cementless and cemented medial unicompartmental knee arthroplasty: results from the Dutch National Arthroplasty Registry. Bone Jt Open. 2024;5(5):401–10. 10.1302/2633-1462.55.BJO-2024-0007.R1 38767223 10.1302/2633-1462.55.BJO-2024-0007.R1 PMC11103876

[43] Fozo ZA, Hussein Ghazal A, Kamal I, et al. A systematic review and network Meta-analysis of the outcomes of patients with total knee arthroplasty using cemented, uncemented, or hybrid techniques. Cureus Published Online Oct. 2023;18. 10.7759/cureus.47299 10.7759/cureus.47299 PMC10589057 37869049

[44] Yang J, Li X, Liu P, Liu X, Li L, Zhang M. The impact of patellofemoral joint diseases on functional outcomes and prosthesis survival in patients undergoing unicompartmental knee arthroplasty: a systematic review and meta-analysis. J Orthop Surg Res. 2024;19(1):840. 10.1186/s13018-024-05273-y 39696549 10.1186/s13018-024-05273-y PMC11656892

[45] Liu Y, Xing Z, Wu B, et al. Association of MRI-based knee osteoarthritis structural phenotypes with short-term structural progression and subsequent total knee replacement. J Orthop Surg Res. 2024;19(1):699. 10.1186/s13018-024-05194-w 39468567 10.1186/s13018-024-05194-w PMC11520466

