# Supplementary material for: Satisfied with the worst health outcomes or unsatisfied with the best: explaining the divergence between good patient-reported outcomes and low satisfaction and vice versa among knee arthroplasty patients – a retrospective cohort study
Source: J Orthop Surg Res. 2025 Jan 23;20:88. doi: 10.1186/s13018-025-05507-7 (PMC11755965; doi:10.1186/s13018-025-05507-7)
Supplement: Supplementary file 1 — Supplementary Material 1 [file 13018_2025_5507_MOESM1_ESM.docx]

**Appendices**

**Multinomial logistic regression: Results Baseline PROs**

| Patient group | Variable | Coef. | | St.Err. | t-value | | p-value | [95% Conf | | Interval] | | Sig |
| --- | --- | --- | --- | --- | --- | --- | --- | --- | --- | --- | --- | --- |
| **Satisfied Achiever**  **(reference group)** |  |  | |  |  | |  |  | |  | |  |
|  |  |  | |  |  | |  |  | |  | |  |
| **Dissatisfied Achiever** | age | .913 | | .104 | -0.79 | | .427 | .73 | | 1.142 | |  |
|  | bmi | .994 | | .018 | -0.35 | | .728 | .958 | | 1.03 | |  |
|  | gender | .993 | | .208 | -0.03 | | .973 | .658 | | 1.498 | |  |
|  | *Education* |  | |  |  | |  |  | |  | |  |
|  | High/middle school degree (reference) | 1 | | . | . | | . | . | | . | |  |
|  | No school degree | 1.842 | | 2.196 | 0.51 | | .608 | .178 | | 19.048 | |  |
|  | Primary school degree | 1.119 | | .28 | 0.45 | | .654 | .685 | | 1.827 | |  |
|  | University degree | .696 | | .198 | -1.27 | | .204 | .398 | | 1.217 | |  |
|  | *Living Situation* |  | |  |  | |  |  | |  | |  |
|  | With partner/family/friends (reference) | 1 | | . | . | | . | . | | . | |  |
|  | living alone | .715 | | .186 | -1.29 | | .197 | .429 | | 1.191 | |  |
|  | In a care facility | 1.57 | | 1.788 | 0.40 | | .692 | .169 | | 14.621 | |  |
|  | other | 3.23 | | 2.349 | 1.61 | | .107 | .777 | | 13.433 | |  |
|  | *Comorbidities* |  | |  |  | |  |  | |  | |  |
|  | Back | 2.317 | | .476 | 4.09 | | 0 | 1.548 | | 3.467 | | *** |
|  | Depression | 1.006 | | .32 | 0.02 | | .986 | .539 | | 1.877 | |  |
|  | Diabetes | 1.659 | | .459 | 1.83 | | .068 | .964 | | 2.855 | | * |
|  | Heart | 1.491 | | .393 | 1.52 | | .129 | .89 | | 2.5 | |  |
|  | Arthritis | 1.405 | | .387 | 1.24 | | .216 | .82 | | 2.41 | |  |
|  | Cancer | .929 | | .355 | -0.19 | | .847 | .439 | | 1.965 | |  |
|  | Stroke | 1.731 | | .801 | 1.18 | | .236 | .698 | | 4.289 | |  |
|  | Blood | 1.094 | | .232 | 0.42 | | .673 | .721 | | 1.659 | |  |
|  | Circulation | 1.921 | | .568 | 2.21 | | .027 | 1.075 | | 3.43 | | ** |
|  | Lung | .983 | | .276 | -0.06 | | .951 | .566 | | 1.705 | |  |
|  | Neurologic | .846 | | .441 | -0.32 | | .749 | .304 | | 2.353 | |  |
|  | *Patient-reported outcomes* |  | |  |  | |  |  | |  | |  |
|  | EQVAS Baseline | .919 | | .105 | -0.74 | | .458 | .734 | | 1.149 | |  |
|  | EQ5D5L Baseline | .896 | | .101 | -0.97 | | .33 | .718 | | 1.117 | |  |
|  | PROMIS-D-SF Baseline | .669 | | .089 | -3.03 | | .002 | .516 | | .867 | | *** |
|  | PROMIS-F-SF Baseline | 1.117 | | .153 | 0.81 | | .418 | .855 | | 1.46 | |  |
|  | Pain Baseline | .954 | | .096 | -0.47 | | .641 | .784 | | 1.162 | |  |
|  | *Treatment variables* |  | |  |  | |  |  | |  | |  |
|  | Rapid recovery | 1 | | .2 | 0.00 | | 1 | .676 | | 1.48 | |  |
|  | Readmission | 1.626 | | .383 | 2.06 | | .039 | 1.024 | | 2.581 | | ** |
|  | Reoperation | 1.797 | | .966 | 1.09 | | .276 | .626 | | 5.156 | |  |
|  | Treating hospital | 1.005 | | .044 | 0.12 | | .905 | .922 | | 1.095 | |  |
|  |  |  | |  |  | |  |  | |  | |  |
|  | Constant | .001 | | .001 | -5.30 | | 0 | 0 | | .013 | | *** |
| **Satisfied Non-achiever** | age | 1.201 | | .131 | 1.68 | | .093 | .97 | | 1.487 | | * |
|  | bmi | 1.031 | | .019 | 1.67 | | .094 | .995 | | 1.068 | | * |
|  | gender | 1.317 | | .26 | 1.40 | | .163 | .895 | | 1.938 | |  |
|  | *Education* |  | |  |  | |  |  | |  | |  |
|  | High/middle school degree (reference) | 1 | | . | . | | . | . | | . | |  |
|  | No school degree | 0 | | .003 | -0.01 | | .993 | 0 | | . | |  |
|  | Primary school degree | 1.014 | | .254 | 0.06 | | .954 | .621 | | 1.656 | |  |
|  | University degree | .755 | | .183 | -1.16 | | .246 | .47 | | 1.214 | |  |
|  | *Living Situation* |  | |  |  | |  |  | |  | |  |
|  | With partner/family/friends (reference) | 1 | | . | . | | . | . | | . | |  |
|  | living alone | .996 | | .241 | -0.02 | | .986 | .619 | | 1.6 | |  |
|  | In a care facility | 0 | | .001 | -0.01 | | .992 | 0 | | . | |  |
|  | other | .785 | | .865 | -0.22 | | .826 | .091 | | 6.803 | |  |
|  | *Comorbidities* |  | |  |  | |  |  | |  | |  |
|  | Back | 1.175 | | .257 | 0.74 | | .46 | .766 | | 1.805 | |  |
|  | Depression | .726 | | .358 | -0.65 | | .516 | .276 | | 1.907 | |  |
|  | Diabetes | 1.148 | | .338 | 0.47 | | .64 | .644 | | 2.044 | |  |
|  | Heart | .989 | | .263 | -0.04 | | .967 | .587 | | 1.667 | |  |
|  | Arthritis | 1.966 | | .574 | 2.31 | | .021 | 1.109 | | 3.485 | | ** |
|  | Cancer | .821 | | .349 | -0.46 | | .643 | .357 | | 1.887 | |  |
|  | Stroke | .819 | | .516 | -0.32 | | .752 | .238 | | 2.817 | |  |
|  | Blood | .894 | | .175 | -0.57 | | .567 | .608 | | 1.313 | |  |
|  | Circulation | 1.31 | | .481 | 0.73 | | .463 | .637 | | 2.692 | |  |
|  | Lung | .951 | | .273 | -0.17 | | .862 | .542 | | 1.67 | |  |
|  | Neurologic | .721 | | .543 | -0.44 | | .663 | .165 | | 3.153 | |  |
|  | *Patient-reported outcomes* |  | |  |  | |  |  | |  | |  |
|  | EQVAS Baseline | .97 | | .107 | -0.28 | | .781 | .782 | | 1.204 | |  |
|  | EQ5D5L Baseline | 1.748 | | .237 | 4.12 | | 0 | 1.34 | | 2.279 | | *** |
|  | PROMIS-D-SF Baseline | .917 | | .125 | -0.64 | | .522 | .702 | | 1.197 | |  |
|  | PROMIS-F-SF Baseline | 1.369 | | .183 | 2.36 | | .018 | 1.054 | | 1.778 | | ** |
|  | Pain Baseline | .891 | | .092 | -1.12 | | .264 | .727 | | 1.091 | |  |
|  | *Treatment variables* |  | |  |  | |  |  | |  | |  |
|  | Rapid recovery | 1.452 | | .271 | 2.00 | | .046 | 1.007 | | 2.092 | | ** |
|  | Readmission | .549 | | .281 | -1.17 | | .242 | .201 | | 1.499 | |  |
|  | Reoperation | 3.044 | | 1.573 | 2.15 | | .031 | 1.105 | | 8.383 | | ** |
|  | Treating hospital | .988 | | .042 | -0.28 | | .78 | .909 | | 1.075 | |  |
|  |  |  | |  |  | |  |  | |  | |  |
|  | Constant | .014 | | .022 | -2.67 | | .008 | .001 | | .322 | | *** |
| **Dissatisfied Non-achiever** | age | .695 | | .08 | -3.15 | | .002 | .554 | | .871 | | *** |
|  | bmi | .977 | | .019 | -1.16 | | .244 | .94 | | 1.016 | |  |
|  | gender | 1.046 | | .219 | 0.21 | | .83 | .694 | | 1.576 | |  |
|  | *Education* |  | |  |  | |  |  | |  | |  |
|  | High/middle school degree (reference) | 1 | | . | . | | . | . | | . | |  |
|  | No school degree | 3.315 | | 3.94 | 1.01 | | .313 | .323 | | 34.055 | |  |
|  | Primary school degree | 1.283 | | .344 | 0.93 | | .352 | .759 | | 2.171 | |  |
|  | University degree | 1.72 | | .413 | 2.26 | | .024 | 1.074 | | 2.755 | | ** |
|  | *Living Situation* |  | |  |  | |  |  | |  | |  |
|  | With partner/family/friends (reference) | 1 | | . | . | | . | . | | . | |  |
|  | living alone | .786 | | .21 | -0.90 | | .367 | .465 | | 1.327 | |  |
|  | In a care facility | 5.633 | | 4.712 | 2.07 | | .039 | 1.093 | | 29.022 | | ** |
|  | other | 2.792 | | 1.987 | 1.44 | | .149 | .692 | | 11.266 | |  |
|  | *Comorbidities* |  | |  |  | |  |  | |  | |  |
|  | Back | 1.885 | | .397 | 3.01 | | .003 | 1.248 | | 2.847 | | *** |
|  | Depression | 1.683 | | .515 | 1.70 | | .089 | .924 | | 3.065 | | * |
|  | Diabetes | 1.105 | | .349 | 0.32 | | .752 | .595 | | 2.051 | |  |
|  | Heart | 1.767 | | .472 | 2.13 | | .033 | 1.046 | | 2.984 | | ** |
|  | Arthritis | 1.406 | | .404 | 1.19 | | .235 | .801 | | 2.47 | |  |
|  | Cancer | .585 | | .274 | -1.14 | | .253 | .233 | | 1.467 | |  |
|  | Stroke | 1.889 | | .888 | 1.35 | | .176 | .752 | | 4.745 | |  |
|  | Blood | 1.124 | | .238 | 0.55 | | .582 | .742 | | 1.701 | |  |
|  | Circulation | 2.431 | | .738 | 2.93 | | .003 | 1.341 | | 4.407 | | *** |
|  | Lung | .815 | | .248 | -0.67 | | .501 | .448 | | 1.48 | |  |
|  | Neurologic | 2.536 | | 1.037 | 2.28 | | .023 | 1.138 | | 5.652 | | ** |
|  | *Patient-reported outcomes* |  | |  |  | |  |  | |  | |  |
|  | EQVAS Baseline | .938 | | .107 | -0.56 | | .573 | .751 | | 1.172 | |  |
|  | EQ5D5L Baseline | 1.047 | | .124 | 0.38 | | .701 | .829 | | 1.321 | |  |
|  | PROMIS-D-SF Baseline | .845 | | .112 | -1.27 | | .206 | .651 | | 1.097 | |  |
|  | PROMIS-F-SF Baseline | 1.009 | | .137 | 0.06 | | .95 | .772 | | 1.317 | |  |
|  | Pain Baseline | 1.037 | | .113 | 0.33 | | .74 | .838 | | 1.283 | |  |
|  | *Treatment variables* |  | |  |  | |  |  | |  | |  |
|  | Rapid recovery | 1.032 | | .207 | 0.16 | | .876 | .696 | | 1.529 | |  |
|  | Readmission | 1.551 | | .388 | 1.75 | | .08 | .949 | | 2.533 | | * |
|  | Reoperation | 2.07 | | 1.091 | 1.38 | | .167 | .737 | | 5.815 | |  |
|  | Treating hospital | 1.005 | | .044 | 0.11 | | .915 | .922 | | 1.095 | |  |
|  |  |  | |  |  | |  |  | |  | |  |
|  | Constant | 0 | | .001 | -5.85 | | 0 | 0 | | .006 | | *** |
|  |  | | | | | | | | | | | |
|  | Mean dependent var | | 1.516 | | | SD dependent var | | | 0.964 | |  |  |
|  | Pseudo r-squared | | 0.085 | | | Number of obs | | | 1546.000 | |  |  |
|  | Chi-square | | 225.874 | | | Prob > chi2 | | | 0.000 | |  |  |
|  | Akaike crit. (AIC) | | 2600.105 | | | Bayesian crit. (BIC) | | | 3081.013 | |  |  |
|  | **** p<.01, ** p<.05, * p<.1* | | | | | | | | | | | |
|  |  | | | | | | | | | | | |

**Multinomial logistic regression: Results M12 PROs**

| Patient group | Variable | Coef. | | St.Err. | t-value | | p-value | [95% Conf | | Interval] | | Sig |
| --- | --- | --- | --- | --- | --- | --- | --- | --- | --- | --- | --- | --- |
| **Satisfied Achiever**  **(reference group)** |  |  | |  |  | |  |  | |  | |  |
| **Dissatisfied Achiever** | age | 1.02 | | .124 | 0.16 | | .872 | .803 | | 1.294 | |  |
|  | bmi | .992 | | .02 | -0.42 | | .672 | .954 | | 1.031 | |  |
|  | gender | .871 | | .191 | -0.63 | | .53 | .566 | | 1.34 | |  |
|  | *Education* |  | |  |  | |  |  | |  | |  |
|  | High/middle school degree (reference) | 1 | | . | . | | . | . | | . | |  |
|  | No school degree | .981 | | 1.365 | -0.01 | | .989 | .064 | | 15.002 | |  |
|  | Primary school degree | 1.007 | | .272 | 0.03 | | .979 | .594 | | 1.708 | |  |
|  | University degree | .71 | | .209 | -1.16 | | .246 | .399 | | 1.265 | |  |
|  | *Living Situation* |  | |  |  | |  |  | |  | |  |
|  | With partner/family/friends (reference) | 1 | | . | . | | . | . | | . | |  |
|  | living alone | .749 | | .209 | -1.04 | | .301 | .433 | | 1.295 | |  |
|  | In a care facility | 1.712 | | 2.096 | 0.44 | | .661 | .155 | | 18.864 | |  |
|  | other | 2.435 | | 1.925 | 1.13 | | .26 | .517 | | 11.468 | |  |
|  | *Comorbidities* |  | |  |  | |  |  | |  | |  |
|  | Back | .98 | | .223 | -0.09 | | .93 | .627 | | 1.532 | |  |
|  | Depression | .74 | | .265 | -0.84 | | .399 | .367 | | 1.491 | |  |
|  | Diabetes | 1.636 | | .486 | 1.66 | | .097 | .914 | | 2.929 | | * |
|  | Heart | 1.203 | | .338 | 0.66 | | .51 | .694 | | 2.086 | |  |
|  | Arthritis | .787 | | .235 | -0.80 | | .422 | .438 | | 1.413 | |  |
|  | Cancer | .721 | | .3 | -0.79 | | .432 | .318 | | 1.632 | |  |
|  | Stroke | 1.47 | | .738 | 0.77 | | .443 | .549 | | 3.933 | |  |
|  | Blood | .92 | | .207 | -0.37 | | .709 | .592 | | 1.428 | |  |
|  | Circulation | 1.597 | | .518 | 1.44 | | .149 | .846 | | 3.015 | |  |
|  | Lung | .857 | | .264 | -0.50 | | .617 | .469 | | 1.568 | |  |
|  | Neurologic | .602 | | .337 | -0.91 | | .365 | .201 | | 1.805 | |  |
|  | *Patient-reported outcomes* |  | |  |  | |  |  | |  | |  |
|  | EQVAS M12 | .766 | | .106 | -1.93 | | .054 | .584 | | 1.005 | | * |
|  | EQ5D5L M12 | .76 | | .109 | -1.92 | | .055 | .574 | | 1.006 | | * |
|  | PROMIS-D-SF M12 | 1.146 | | .161 | 0.97 | | .33 | .871 | | 1.509 | |  |
|  | PROMIS-F-SF M12 | .615 | | .096 | -3.12 | | .002 | .453 | | .835 | | *** |
|  | Pain M12 | .466 | | .061 | -5.88 | | 0 | .361 | | .601 | | *** |
|  | *Treatment variables* |  | |  |  | |  |  | |  | |  |
|  | Rapid recovery | .895 | | .188 | -0.53 | | .596 | .593 | | 1.349 | |  |
|  | Readmission | 1.616 | | .41 | 1.89 | | .059 | .983 | | 2.657 | | * |
|  | Reoperation | 1.195 | | .672 | 0.32 | | .751 | .397 | | 3.6 | |  |
|  | Treating hospital | 1.02 | | .048 | 0.42 | | .673 | .931 | | 1.117 | |  |
|  |  |  | |  |  | |  |  | |  | |  |
|  | Constant | .077 | | .111 | -1.78 | | .075 | .005 | | 1.295 | | * |
| **Satisfied Non-achiever** | age | 1.446 | | .163 | 3.27 | | .001 | 1.16 | | 1.804 | | *** |
|  | bmi | 1.01 | | .019 | 0.52 | | .601 | .974 | | 1.047 | |  |
|  | gender | 1.58 | | .317 | 2.28 | | .023 | 1.066 | | 2.342 | | ** |
|  | *Education* |  | |  |  | |  |  | |  | |  |
|  | High/middle school degree (reference) | 1 | | . | . | | . | . | | . | |  |
|  | No school degree | 0 | | .001 | -0.01 | | .991 | 0 | | . | |  |
|  | Primary school degree | .893 | | .229 | -0.44 | | .658 | .54 | | 1.476 | |  |
|  | University degree | .748 | | .185 | -1.18 | | .24 | .461 | | 1.214 | |  |
|  | *Living Situation* |  | |  |  | |  |  | |  | |  |
|  | With partner/family/friends (reference) | 1 | | . | . | | . | . | | . | |  |
|  | living alone | 1.042 | | .261 | 0.16 | | .871 | .637 | | 1.703 | |  |
|  | In a care facility | 0 | | .001 | -0.01 | | .99 | 0 | | . | |  |
|  | other | .691 | | .802 | -0.32 | | .75 | .071 | | 6.719 | |  |
|  | *Comorbidities* |  | |  |  | |  |  | |  | |  |
|  | Back | .474 | | .112 | -3.17 | | .002 | .298 | | .752 | | *** |
|  | Depression | .329 | | .168 | -2.17 | | .03 | .121 | | .896 | | ** |
|  | Diabetes | 1.122 | | .341 | 0.38 | | .704 | .619 | | 2.036 | |  |
|  | Heart | .777 | | .217 | -0.90 | | .366 | .45 | | 1.342 | |  |
|  | Arthritis | .834 | | .255 | -0.59 | | .553 | .458 | | 1.519 | |  |
|  | Cancer | .513 | | .228 | -1.51 | | .132 | .215 | | 1.224 | |  |
|  | Stroke | .568 | | .364 | -0.88 | | .377 | .162 | | 1.991 | |  |
|  | Blood | .773 | | .156 | -1.28 | | .202 | .52 | | 1.148 | |  |
|  | Circulation | .965 | | .361 | -0.10 | | .924 | .463 | | 2.011 | |  |
|  | Lung | 1 | | .297 | -0.00 | | .999 | .558 | | 1.79 | |  |
|  | Neurologic | .385 | | .294 | -1.25 | | .211 | .086 | | 1.716 | |  |
|  | *Patient-reported outcomes* |  | |  |  | |  |  | |  | |  |
|  | EQVAS M12 | .764 | | .103 | -1.99 | | .046 | .586 | | .996 | | ** |
|  | EQ5D5L M12 | .71 | | .108 | -2.25 | | .025 | .526 | | .957 | | ** |
|  | PROMIS-D-SF M12 | 1.15 | | .157 | 1.02 | | .307 | .88 | | 1.503 | |  |
|  | PROMIS-F-SF M12 | .718 | | .105 | -2.27 | | .023 | .539 | | .955 | | ** |
|  | Pain M12 | .547 | | .07 | -4.73 | | 0 | .426 | | .702 | | *** |
|  | *Treatment variables* |  | |  |  | |  |  | |  | |  |
|  | Rapid recovery | 1.418 | | .272 | 1.82 | | .068 | .974 | | 2.065 | | * |
|  | Readmission | .611 | | .301 | -1.00 | | .317 | .232 | | 1.605 | |  |
|  | Reoperation | 2.075 | | 1.121 | 1.35 | | .176 | .72 | | 5.98 | |  |
|  | Treating hospital | 1.007 | | .044 | 0.16 | | .875 | .924 | | 1.098 | |  |
|  |  |  | |  |  | |  |  | |  | |  |
|  | Constant | 5.5 | | 9.169 | 1.02 | | .307 | .21 | | 144.345 | |  |
| **Dissatisfied Non-achiever** | age | .821 | | .112 | -1.45 | | .147 | .629 | | 1.072 | |  |
|  | bmi | .94 | | .022 | -2.63 | | .009 | .898 | | .984 | | *** |
|  | gender | 1.243 | | .307 | 0.88 | | .378 | .766 | | 2.019 | |  |
|  | *Education* |  | |  |  | |  |  | |  | |  |
|  | High/middle school degree (reference) | 1 | | . | . | | . | . | | . | |  |
|  | No school degree | .296 | | .509 | -0.71 | | .479 | .01 | | 8.62 | |  |
|  | Primary school degree | 1.081 | | .356 | 0.24 | | .814 | .566 | | 2.061 | |  |
|  | University degree | 2.365 | | .674 | 3.02 | | .003 | 1.353 | | 4.135 | | *** |
|  | *Living Situation* |  | |  |  | |  |  | |  | |  |
|  | With partner/family/friends (reference) | 1 | | . | . | | . | . | | . | |  |
|  | living alone | .885 | | .282 | -0.38 | | .702 | .474 | | 1.654 | |  |
|  | In a care facility | 3.695 | | 4.535 | 1.06 | | .287 | .333 | | 40.954 | |  |
|  | other | 1.757 | | 1.6 | 0.62 | | .536 | .295 | | 10.465 | |  |
|  | *Comorbidities* |  | |  |  | |  |  | |  | |  |
|  | Back | .463 | | .125 | -2.85 | | .004 | .273 | | .786 | | *** |
|  | Depression | .821 | | .322 | -0.50 | | .615 | .38 | | 1.771 | |  |
|  | Diabetes | 1.14 | | .428 | 0.35 | | .728 | .546 | | 2.379 | |  |
|  | Heart | 1.31 | | .423 | 0.84 | | .403 | .696 | | 2.465 | |  |
|  | Arthritis | .543 | | .194 | -1.71 | | .088 | .269 | | 1.095 | | * |
|  | Cancer | .316 | | .177 | -2.05 | | .04 | .105 | | .95 | | ** |
|  | Stroke | 1.631 | | .915 | 0.87 | | .383 | .543 | | 4.897 | |  |
|  | Blood | .785 | | .198 | -0.96 | | .339 | .479 | | 1.289 | |  |
|  | Circulation | 1.539 | | .588 | 1.13 | | .26 | .727 | | 3.256 | |  |
|  | Lung | .555 | | .211 | -1.55 | | .122 | .263 | | 1.169 | |  |
|  | Neurologic | 1.376 | | .699 | 0.63 | | .53 | .508 | | 3.724 | |  |
|  | *Patient-reported outcomes* |  | |  |  | |  |  | |  | |  |
|  | EQVAS M12 | .748 | | .115 | -1.90 | | .058 | .554 | | 1.01 | | * |
|  | EQ5D5L M12 | .403 | | .058 | -6.31 | | 0 | .304 | | .535 | | *** |
|  | PROMIS-D-SF M12 | .893 | | .143 | -0.71 | | .48 | .652 | | 1.223 | |  |
|  | PROMIS-F-SF M12 | .844 | | .153 | -0.94 | | .348 | .592 | | 1.203 | |  |
|  | Pain M12 | .452 | | .064 | -5.64 | | 0 | .343 | | .596 | | *** |
|  | *Treatment variables* |  | |  |  | |  |  | |  | |  |
|  | Rapid recovery | 1.013 | | .239 | 0.05 | | .956 | .638 | | 1.609 | |  |
|  | Readmission | 1.451 | | .443 | 1.22 | | .222 | .798 | | 2.639 | |  |
|  | Reoperation | 1.397 | | .79 | 0.59 | | .554 | .461 | | 4.231 | |  |
|  | Treating hospital | 1.037 | | .055 | 0.67 | | .5 | .934 | | 1.151 | |  |
|  |  |  | |  |  | |  |  | |  | |  |
|  | Constant | .807 | | 1.256 | -0.14 | | .891 | .038 | | 17.045 | |  |
|  |  | | | | | | | | | | | |
|  | Mean dependent var | | 1.516 | | | SD dependent var | | | 0.964 | |  |  |
|  | Pseudo r-squared | | 0.223 | | | Number of obs | | | 1546.000 | |  |  |
|  | Chi-square | | 588.901 | | | Prob > chi2 | | | 0.000 | |  |  |
|  | Akaike crit. (AIC) | | 2237.077 | | | Bayesian crit. (BIC) | | | 2717.986 | |  |  |
|  | **** p<.01, ** p<.05, * p<.1* | | | | | | | | | | | |
|  |  | | | | | | | | | | | |

**Multinomial logistic regression: Change in PROs**

| Patient group |  | Coef. | | St.Err. | t-value | | p-value | [95% Conf | | Interval] | | Sig |
| --- | --- | --- | --- | --- | --- | --- | --- | --- | --- | --- | --- | --- |
| **Satisfied Achiever**  **(reference group)** |  |  | |  |  | |  |  | |  | |  |
| **Dissatisfied Achiever** | age | .871 | | .103 | -1.17 | | .241 | .691 | | 1.097 | |  |
|  | bmi | 1.012 | | .019 | 0.60 | | .546 | .974 | | 1.051 | |  |
|  | gender | .742 | | .158 | -1.40 | | .163 | .489 | | 1.128 | |  |
|  | *Education* |  | |  |  | |  |  | |  | |  |
|  | High/middle school degree (reference) | 1 | | . | . | | . | . | | . | |  |
|  | No school degree | 3.156 | | 4.005 | 0.91 | | .365 | .262 | | 37.962 | |  |
|  | Primary school degree | 1.185 | | .309 | 0.65 | | .516 | .71 | | 1.977 | |  |
|  | University degree | .607 | | .176 | -1.72 | | .086 | .344 | | 1.073 | | * |
|  | *Living Situation* |  | |  |  | |  |  | |  | |  |
|  | With partner/family/friends (reference) | 1 | | . | . | | . | . | | . | |  |
|  | living alone | .802 | | .217 | -0.81 | | .415 | .472 | | 1.364 | |  |
|  | In a care facility | 1.114 | | 1.442 | 0.08 | | .934 | .088 | | 14.087 | |  |
|  | other | 3.282 | | 2.502 | 1.56 | | .119 | .737 | | 14.618 | |  |
|  | *Comorbidities* |  | |  |  | |  |  | |  | |  |
|  | Back | 2.098 | | .435 | 3.57 | | 0 | 1.397 | | 3.152 | | *** |
|  | Depression | 1.354 | | .439 | 0.94 | | .35 | .717 | | 2.556 | |  |
|  | Diabetes | 1.996 | | .585 | 2.36 | | .018 | 1.124 | | 3.544 | | ** |
|  | Heart | 1.403 | | .379 | 1.25 | | .21 | .826 | | 2.381 | |  |
|  | Arthritis | 1.341 | | .381 | 1.03 | | .302 | .768 | | 2.341 | |  |
|  | Cancer | .882 | | .354 | -0.31 | | .754 | .402 | | 1.935 | |  |
|  | Stroke | 1.672 | | .813 | 1.06 | | .291 | .644 | | 4.338 | |  |
|  | Blood | 1.046 | | .23 | 0.21 | | .837 | .68 | | 1.608 | |  |
|  | Circulation | 2.252 | | .699 | 2.62 | | .009 | 1.226 | | 4.139 | | *** |
|  | Lung | .929 | | .272 | -0.25 | | .802 | .523 | | 1.65 | |  |
|  | Neurologic | .917 | | .502 | -0.16 | | .875 | .314 | | 2.682 | |  |
|  | *Patient-reported outcomes* |  | |  |  | |  |  | |  | |  |
|  | EQVAS ∆ | .761 | | .09 | -2.31 | | .021 | .603 | | .959 | | ** |
|  | EQ5D5L ∆ | .978 | | .115 | -0.19 | | .85 | .777 | | 1.231 | |  |
|  | PROMIS-D-SF ∆ | 1.236 | | .149 | 1.77 | | .077 | .977 | | 1.565 | | * |
|  | PROMIS-F-SF ∆ | .611 | | .078 | -3.83 | | 0 | .476 | | .786 | | *** |
|  | Pain ∆ | .503 | | .059 | -5.89 | | 0 | .4 | | .632 | | *** |
|  | *Treatment variables* |  | |  |  | |  |  | |  | |  |
|  | Rapid recovery | .951 | | .196 | -0.24 | | .808 | .636 | | 1.424 | |  |
|  | Readmission | 1.536 | | .381 | 1.73 | | .084 | .944 | | 2.497 | | * |
|  | Reoperation | 1.437 | | .805 | 0.65 | | .518 | .479 | | 4.307 | |  |
|  | Treating hospital | 1.004 | | .046 | 0.10 | | .924 | .918 | | 1.099 | |  |
|  |  |  | |  |  | |  |  | |  | |  |
|  | Constant | .001 | | .001 | -5.32 | | 0 | 0 | | .011 | | *** |
| **Satisfied Non-achiever** | age | 1.233 | | .142 | 1.82 | | .069 | .984 | | 1.545 | | * |
|  | bmi | 1.033 | | .02 | 1.68 | | .092 | .995 | | 1.073 | | * |
|  | gender | 1.138 | | .237 | 0.62 | | .534 | .757 | | 1.711 | |  |
|  | *Education* |  | |  |  | |  |  | |  | |  |
|  | High/middle school degree (reference) | 1 | | . | . | | . | . | | . | |  |
|  | No school degree | 0 | | .002 | -0.01 | | .988 | 0 | | . | |  |
|  | Primary school degree | 1.045 | | .281 | 0.16 | | .871 | .617 | | 1.769 | |  |
|  | University degree | .694 | | .178 | -1.43 | | .154 | .42 | | 1.146 | |  |
|  | *Living Situation* |  | |  |  | |  |  | |  | |  |
|  | With partner/family/friends (reference) | 1 | | . | . | | . | . | | . | |  |
|  | living alone | 1.12 | | .291 | 0.44 | | .661 | .674 | | 1.863 | |  |
|  | In a care facility | 0 | | .001 | -0.01 | | .989 | 0 | | . | |  |
|  | other | .609 | | .72 | -0.42 | | .675 | .06 | | 6.183 | |  |
|  | *Comorbidities* |  | |  |  | |  |  | |  | |  |
|  | Back | .773 | | .178 | -1.12 | | .263 | .493 | | 1.213 | |  |
|  | Depression | .462 | | .238 | -1.50 | | .135 | .168 | | 1.27 | |  |
|  | Diabetes | 1.411 | | .445 | 1.09 | | .275 | .76 | | 2.62 | |  |
|  | Heart | .806 | | .228 | -0.76 | | .446 | .462 | | 1.404 | |  |
|  | Arthritis | 1.22 | | .386 | 0.63 | | .53 | .656 | | 2.27 | |  |
|  | Cancer | .55 | | .253 | -1.30 | | .193 | .223 | | 1.354 | |  |
|  | Stroke | .781 | | .512 | -0.38 | | .706 | .216 | | 2.823 | |  |
|  | Blood | .786 | | .166 | -1.14 | | .254 | .52 | | 1.188 | |  |
|  | Circulation | 1.222 | | .479 | 0.51 | | .609 | .567 | | 2.633 | |  |
|  | Lung | .778 | | .241 | -0.81 | | .419 | .424 | | 1.429 | |  |
|  | Neurologic | .58 | | .459 | -0.69 | | .491 | .123 | | 2.73 | |  |
|  | *Patient-reported outcomes* |  | |  |  | |  |  | |  | |  |
|  | EQVAS ∆ | .761 | | .09 | -2.30 | | .022 | .603 | | .961 | | ** |
|  | EQ5D5L ∆ | .515 | | .069 | -4.98 | | 0 | .396 | | .669 | | *** |
|  | PROMIS-D-SF ∆ | .973 | | .122 | -0.22 | | .827 | .761 | | 1.244 | |  |
|  | PROMIS-F-SF ∆ | .509 | | .066 | -5.24 | | 0 | .395 | | .656 | | *** |
|  | Pain ∆ | .6 | | .07 | -4.40 | | 0 | .478 | | .754 | | *** |
|  | *Treatment variables* |  | |  |  | |  |  | |  | |  |
|  | Rapid recovery | 1.419 | | .282 | 1.76 | | .078 | .961 | | 2.094 | | * |
|  | Readmission | .489 | | .253 | -1.38 | | .167 | .177 | | 1.35 | |  |
|  | Reoperation | 2.078 | | 1.131 | 1.34 | | .179 | .715 | | 6.04 | |  |
|  | Treating hospital | 1.004 | | .046 | 0.08 | | .936 | .917 | | 1.098 | |  |
|  |  |  | |  |  | |  |  | |  | |  |
|  | Constant | .292 | | .481 | -0.75 | | .455 | .012 | | 7.359 | |  |
| **Dissatisfied Non-achiever** | age | .65 | | .085 | -3.30 | | .001 | .504 | | .84 | | *** |
|  | bmi | .976 | | .022 | -1.05 | | .292 | .933 | | 1.021 | |  |
|  | gender | .77 | | .188 | -1.07 | | .285 | .476 | | 1.244 | |  |
|  | *Education* |  | |  |  | |  |  | |  | |  |
|  | High/middle school degree (reference) | 1 | | . | . | | . | . | | . | |  |
|  | No school degree | 1.183 | | 2.503 | 0.08 | | .937 | .019 | | 74.875 | |  |
|  | Primary school degree | 1.298 | | .422 | 0.80 | | .422 | .687 | | 2.455 | |  |
|  | University degree | 2.005 | | .561 | 2.48 | | .013 | 1.158 | | 3.471 | | ** |
|  | *Living Situation* |  | |  |  | |  |  | |  | |  |
|  | With partner/family/friends (reference) | 1 | | . | . | | . | . | | . | |  |
|  | living alone | 1.086 | | .335 | 0.27 | | .788 | .594 | | 1.987 | |  |
|  | In a care facility | 2.959 | | 3.114 | 1.03 | | .303 | .376 | | 23.28 | |  |
|  | other | 1.51 | | 1.446 | 0.43 | | .667 | .231 | | 9.859 | |  |
|  | *Comorbidities* |  | |  |  | |  |  | |  | |  |
|  | Back | 1.135 | | .282 | 0.51 | | .609 | .698 | | 1.846 | |  |
|  | Depression | 1.563 | | .579 | 1.21 | | .227 | .757 | | 3.23 | |  |
|  | Diabetes | 1.364 | | .521 | 0.81 | | .416 | .645 | | 2.885 | |  |
|  | Heart | 1.516 | | .475 | 1.33 | | .185 | .82 | | 2.803 | |  |
|  | Arthritis | .947 | | .331 | -0.16 | | .877 | .477 | | 1.88 | |  |
|  | Cancer | .464 | | .252 | -1.41 | | .158 | .16 | | 1.348 | |  |
|  | Stroke | 1.884 | | 1.059 | 1.13 | | .26 | .626 | | 5.668 | |  |
|  | Blood | .861 | | .215 | -0.60 | | .551 | .528 | | 1.406 | |  |
|  | Circulation | 2.564 | | .959 | 2.52 | | .012 | 1.232 | | 5.336 | | ** |
|  | Lung | .593 | | .221 | -1.40 | | .16 | .286 | | 1.229 | |  |
|  | Neurologic | 3.534 | | 1.743 | 2.56 | | .01 | 1.344 | | 9.292 | | ** |
|  | *Patient-reported outcomes* |  | |  |  | |  |  | |  | |  |
|  | EQVAS ∆ | .718 | | .1 | -2.38 | | .017 | .547 | | .943 | | ** |
|  | EQ5D5L ∆ | .415 | | .062 | -5.91 | | 0 | .31 | | .555 | | *** |
|  | PROMIS-D-SF ∆ | .765 | | .106 | -1.93 | | .053 | .583 | | 1.004 | | * |
|  | PROMIS-F-SF ∆ | .658 | | .095 | -2.89 | | .004 | .496 | | .874 | | *** |
|  | Pain ∆ | .338 | | .046 | -7.90 | | 0 | .258 | | .442 | | *** |
|  | *Treatment variables* |  | |  |  | |  |  | |  | |  |
|  | Rapid recovery | .998 | | .231 | -0.01 | | .993 | .633 | | 1.572 | |  |
|  | Readmission | 1.392 | | .384 | 1.20 | | .229 | .812 | | 2.389 | |  |
|  | Reoperation | 1.731 | | .969 | 0.98 | | .327 | .578 | | 5.187 | |  |
|  | Treating hospital | 1.035 | | .054 | 0.66 | | .508 | .934 | | 1.147 | |  |
|  |  |  | |  |  | |  |  | |  | |  |
|  | Constant | .002 | | .002 | -4.39 | | 0 | 0 | | .029 | | *** |
|  |  | | | | | | | | | | | |
|  | Mean dependent var | | 1.516 | | | SD dependent var | | | 0.964 | |  |  |
|  | Pseudo r-squared | | 0.236 | | | Number of obs | | | 1546.000 | |  |  |
|  | Chi-square | | 624.369 | | | Prob > chi2 | | | 0.000 | |  |  |
|  | Akaike crit. (AIC) | | 2201.610 | | | Bayesian crit. (BIC) | | | 2682.518 | |  |  |
|  | **** p<.01, ** p<.05, * p<.1* | | | | | | | | | | | |
|  |  | | | | | | | | | | | |
